# Research on the Development of Theme Trends and Changes of Knowledge Structures of Drug Therapy Studies on Major Depressive Disorder Since the 21^st^ Century: A Bibliometric Analysis

**DOI:** 10.3389/fpsyt.2020.00647

**Published:** 2020-07-10

**Authors:** Li Duan, Yunfeng Gao, Xiaojun Shao, ChunSheng Tian, Chunfeng Fu, Gang Zhu

**Affiliations:** ^1^ Department of Psychiatry, The First Affiliated Hospital of China Medical University, Shenyang, China; ^2^ School of Nursing, Chengde Medical University, Chengde, China; ^3^ Department of Hand and Foot Surgery, The Affiliated Hospital of Chengde Medical University, Chengde, China; ^4^ Central Laboratory, The First Affiliated Hospital of China Medical University, Shenyang, China

**Keywords:** bibliometric analysis, co-occurrence analysis, drug therapy, major depressive disorder, social network analysis, strategic diagram

## Abstract

**Background:**

Antidepressant treatment is one of the most effective ways of relieving or curing depressive symptoms in patients with major depressive disorder (MDD). Although many studies have explored the efficacy, tolerability, adverse reactions, and functional mechanism of the disease, there has been no systematic evaluation of the relevant results in this field.

**Aim:**

This paper aims to analyze the theme trends and knowledge structure of drug therapy studies on MDD since the 21^st^ century by employing bibliometric analysis.

**Methods:**

Literature published in PubMed and related to drug therapy studies on MDD were retrieved between 2001 and 2018 in 6-year increments. After extracting major Medical Subject Headings (MeSH) terms/MeSH subheadings, bi-clustering analysis, social network analysis, and strategic diagrams were employed to complete bibliometric analysis.

**Results:**

Overall, 1,577, 2,680, 2,848 relevant research articles were retrieved for the periods during 2001–2006, 2007–2012, and 2013–2018, respectively. In line with strategic diagrams, the main undeveloped and peripheral theme clusters during 2001–2006 were functional mechanisms of antidepressants in pathophysiology, neuroendocrinology and neural biochemistry. These themes were replaced during 2007–2012 by clinical efficacy and influencing factors of antidepressants with or without psychotherapy, mechanisms of adverse reactions of antidepressants, and predictive studies of clinical therapeutic effects of antidepressants based on brain imaging. During 2013–2018 application and evaluation of new antidepressant agents, early identification and prevention of suicide of patients with MDD, as well as genetic- or bio-markers affecting the response and efficacy of antidepressants were the primary themes. Based on social network analyses, emerging hotspots, such as antidepressive agents, second-generation/adverse effects, depressive disorder, major/metabolism, psychotherapy/methods, and brain/drug effects could be identified during 2007–2012 and 2013–2018.

**Conclusions:**

These undeveloped theme clusters and emerging hotspots can be helpful for researchers to clarify the current status of their respective fields and future trends, and to generate novel ideas that may launch new research directions.

## Introduction

Major depressive disorder (MDD), also termed as clinical/unipolar depressive disorder, affects more than 264 million people worldwide. It is considered to be the most common psychiatric/mental disorder and is characterized by complex and widely various physical, psychological, and behavioral symptoms in individuals (especially for adolescent and elderly groups) (1). Epidemiological investigations reported the life-time prevalence of MDD in different countries, such as the United States, 16.2% (2), Canada, 11.3% (3), and China, 3.4% (4), with considerable regional variation. As a chronic mental disorder, MDD exerts a substantial burden on patients (e.g., cognitive impairment, premature mortality, and even suicide) (5, 6), their families (e.g., physical and economic burden) (7), and society (e.g., the economic burden of MDD in US is estimated to exceed $210 billion) (8). Moreover, it is reported that psychiatric disorders account for 22.8% of the global burden of disease, among which MDD is the main cause of disability, and this proportion will increase along with population growth and ageing (9). World Health Organization (WHO) has ranked MDD as the second leading cause of disability globally, and predicts it to be one of the top three leading causes of disease burden in high-income countries by 2030, second only to HIV/AIDS (10).

In the early 20^th^ century, the term “psychopharmacology” started to emerge, and it was not until the 1950s and 1960s that exploration and clinical implementation of medicinal antidepressants occurred, respectively (11). Evidence shows that successful antidepressant treatment is one of the most effective ways of reducing disability, preventing morbidity, and improving quality of life (QOL) for patients with MDD (12). However, due to the complex etiology and pathogenesis of depressive disorders, the mechanism of action of antidepressants are mostly based on the monoamine hypothesis mainly involving serotonin, norepinephrine, and dopamine and its receptor (13). At present, more than 30 antidepressant drugs are available around the world, of which at least 20 are regularly taken by adult patients with depressive disorders (14). Categories of antidepressant drugs commonly used in clinic are first generation antidepressant drugs developed during the “golden decade” of psychopharmacology in the 1950s, and mainly included tricyclic antidepressants and monoamine oxidase inhibitors. Second generation antidepressant drugs, developed in the late 1980s and early 1990s, are generally considered to be the new generation of clinical treatment for MDD and are represented by selective serotonin reuptake inhibitors (SSRIs) and serotonin-norepinephrine reuptake inhibitors (SNRIs) (11).

In addition, given that a significant number of patients fail to respond to the existing above-mentioned antidepressant agents, the development and application of atypical antidepressants (15), phytochemicals (16), and new drug therapeutic targets (17), as well as the combination of medication and other psychotherapy (e.g., cognitive behavior therapy) (18) have become one of the important research directions in the treatment of depressive disorder. Moreover, based on the utilization of antidepressant drugs, scholars also compared the clinical treatment effectiveness by employing outcome indicators, such as acceptability, efficacy, tolerance, adverse reactions, and changes in brain structure and function (19–21).

Thus, scholars utilized MDD and drug therapy as a research subject to carry out large numbers of clinical and basic studies, which provided significant results. At the same time, the quality of papers published in influential journals increased, and the publications showed an increasing trend year by year. This increase in publications in turn caused scholars to begin to devote more energy analyzing the current frontiers and research hotspots in their own respective fields. The term “bibliometrics” was first proposed by the British scholar Pritchard in 1969 (22), which was defined as “that knowledge structure and development could be generated and organized through information processing by combining mathematical and statistical methods” (23, 24). With the gradual maturity of bibliometrics research, its application has been expanded to many fields, such as social (25) and medical sciences (26). Moreover, according to carry out scientific and standardized management, as well as bibliometric, citation, and quantitative analysis of the information resources, scholars understand the current status and dynamic trends of the related discipline or research field. Co-word analysis, as a significant branch of bibliometric analysis, first appeared in the 1970s and was elaborated in the article written by Callon (27). Statistical methods in co-word analysis are mostly related to social network analysis (SNA), clustering analysis, and strategic diagram.

Here, we comprehensively adopt the methods of co-word analysis to conduct quantitative statistical analysis and qualitative standardized discussion on publications related to drug therapy studies on MDD from 2001 to 2018, and aim to gain insights into hotspots, knowledge structure, and theme trends in this field, as well as to guide clinicians and researchers to address clinical treatment and basic research gaps.

## Methods

### Data Sources and Bibliographic Matrix Setup

PubMed, as a free search engine and a part of the Entrez information retrieval system, was developed by National Center for Biotechnology Information of the National Library of Medicine in 2000. It provides a database of biomedical manuscript and abstract searches, with the central theme of medicine, but also other medically related fields by employing search strategies of Medical Subject Headings (MeSH) and text-word searches. MeSH is a typical normative language, which standardizes synonyms of the same concept to ensure the correspondence between words and concepts. In the actual implementation of the retrieval strategy, the application of MeSH retrieval can effectively improve the recall ratio and precision ratio of the retrieval results.

In this study, publications used in bibliometric analyses were retrieved and downloaded from PubMed by employing the retrieval strategy of [Search ((((“Depressive Disorder, Major”[MeSH]) OR “Major depressive disorder”[Title/Abstract]) OR “MDD”[Title/Abstract])) AND ((“Drug therapy”[MeSH]) OR “Antidepressive Agents” [MeSH]) Filters: Journal Article[article type]]. In addition, in order to dynamically analyze the changes in high frequency major MeSH terms/MeSH subheadings and to develop trends and changes of knowledge structure for drug therapy studies on MDD, the publication data were divided into three periods, including January 1, 2001 to December 31, 2006, January 1, 2007 to December 31, 2012, and January 1, 2013 to December 31, 2018. Analysis parameters, including distribution of countries, titles of the periodicals and papers, publication quantity, and publication years, as well as author names and keywords, were accurately extracted, downloaded and properly saved in an XML format. The literature screening was completed by two researchers independently by evaluating and excluding irrelevant articles. The concordance rate (the value of Kappa coefficient) between them was 0.95, indicating a strong agreement (28). The inclusion criteria for articles were all studies must be primarily focus on drug therapy for MDD; article type was classified as journal article. Exclusion criteria were: studies which take “bipolar disorder” as major MeSH terms and “therapy, drug therapy, pathology, physiopathology, blood, genetics” as MeSH subheadings; publication time was not during the retrieval time limit; published as editorial, comments, or conference paper. Ultimately, 1,577, 2,680, 2,848 articles were included in each period and were used to complete the subsequently statistics.

H-index was first proposed by Jorge Hirsch (29) to quantify the work of individual researchers, and was gradually expanded to evaluate the influence of patents (30), academic journals (31), research institutions (32), and bibliometric analysis (33). Its original definition was expounded by incorporating both quantity and quality; “A scientist has index *h* if *h* of his or her *N_p_* papers has at least *h* citations each and the other (*N_p_-h*) papers have ≤ *h* citation each” where *N_p_* stands for the number of papers published over *n* years.

In the current study, we use the above concept to implement a number of steps: ① Bibliographic information, such as major MeSH terms and major MeSH subheadings were extracted by employing the bibliographic item co-occurrence matrix system (BICOMBS) (34). ② According to word frequency statistics, major MeSH terms/MeSH subheadings were arranged in descending order. When the word frequency of the MeSH terms is the same as its rank number, then this number is the threshold value of high- and low-frequency major MeSH terms/MeSH subheadings. In reality, the MeSH terms’ rank number is not always the same as its frequency, and we often take the MeSH term(s) whose occurrence frequency is one less than the rank of the high-frequency Mesh terms so as not to overlook them.

In addition, matrices of the term-source literature and the term-term co-occurrence were generated by the bibliographic item co-occurrence matrix system to prepare for bi-clustering, strategic diagram, and social network analyses.

### Bi-Clustering Analysis of High-Frequency MeSH Terms/MeSH Subheadings

Bi-clustering, also known as co-clustering, bi-dimensional clustering, two-way clustering, or subspace clustering, was introduced by Hartigan in 1970s (35). It refers to simultaneously clustering rows and columns of a data matrix. In recent years, it has been widely used to implement data analysis in the fields, including gene expression (36) and cancer immunotherapy (37). After co-word analysis of these extracted high-frequency major MeSH terms/MeSH subheadings, we inputted three matrices of term-source literature into gCLUTO 1.0 (Graphical Clustering Toolkit, Version 1.0, which was designed as a graphical front-end for the CLUTO data clustering library by Rasmussen, et al.) (38) for bi-clustering analysis by setting the parameters as repeated bisection for clustering, cosine function for similarity calculation, and I^2^ for clustering standard function.

In addition, representative literature that contributes to the formation of the clusters can be extracted by two parameters, including descriptive (literatures that represents this class of characteristics) and discriminating (literature that distinguishes it from other clusters) features. Moreover, we can also trace the original literature from the PubMed database on the basis of their PubMed Unique Identifier, which was extracted by gCLUTO and were significant for analyzing and summarizing the content of each cluster.

### Strategic Diagram Analysis

In 1988, Law et al. (39) firstly proposed the statistical analysis method of strategic diagram. As a significant branch of co-word analysis, it is mainly used to describe the internal relations and mutual influences so as to further predict the theme trends in a certain research field. After more than 30 years of development, strategic diagram has been applied in various disciplines, such as information science (40), higher education (41), and medicine (42), among others. As seen in **Figure 6(i)**, strategic diagram is a two-dimensional coordinate axis, in which X-axis represents centrality, indicating the intensity of the interaction between clusters, and Y-axis represents density, indicating the strength of the connections of hotspots within a theme cluster (43). In general, the two above indices can be calculated on the basis of a term-term matrix. In addition, according to the coordinate position of each cluster in the strategic diagram, we can analyze its development and evolution trend.

In the specific implementation of statistical analysis, GraphPad Prism 5.0 software (GraphPad, Inc., La Jolla, CA, USA) was employed to plot a strategic diagram in each period from 2001 to 2018.

### Social Network Analysis

SNA originated in early 20^th^ century and refers to a method of studying social interactions and the structure of these interactions among social factors (e.g., individuals, groups and organizations) (44). Though it has been widely applied in the field of sociology as a paradigm of social science research, it is now used across multiple disciplines (45, 46), including organizational psychology, biology, and communications.

In this study, the SNA was built for each period to explore the knowledge structure and to predict the theme trends of drug therapy studies on MDD on the basis of construction of a term-term co-occurrence matrix. All calculations concerning the SNA were carried out by using UCINET Version 6.0 (University of California at Irvine Network, Version 6.0), which is a software tool for analysis and visualization of exploratory data network and belongs to Analytic Technologies Co. (Nicholasville, Kentucky, USA). The visualization of the network structure was demonstrated by employing NetDraw 2.084 software, which was developed by Steve Borgatti (http://www.analytictech.com/downloadnd.htm). In addition, to objectively evaluate the importance of each node within the SNA in each period, three involved centrality parameters were computed and analyzed as follows:

- *Degree centrality* is arguably the simplest conceptually and can be used to measure how well-connected a given node is, in other words, it can be obtained by calculating how many direct connections the given node has with others within a network (47). Nodes with higher value of degree centrality have agonistic interaction with many other nodes, thus, it is suitable for evaluating the co-occurrence level among nodes.
- *Betweenness centrality* can be used to measures the extent to which the given node lies on the shortest paths between two other nodes of a network. Nodes with higher value of betweenness centrality are important for controlling the connections within a network, especially if they serve as a bridge or cut point between two network components (48).
- *Closeness centrality* can be used to evaluate how close the given node is to all other nodes in a network. The calculation process of this centrality parameter is based on the inverse of the shortest path lengths between the given node and other nodes within a network. That is, the more central the given node is, the larger its closeness centrality value and the closer it is to all the other nodes (44).

Considering that betweenness centrality is more suitable for describing the decisive role in the whole network, we selected it to scale the size of the nodes in the SNA.

**Figure 1:**
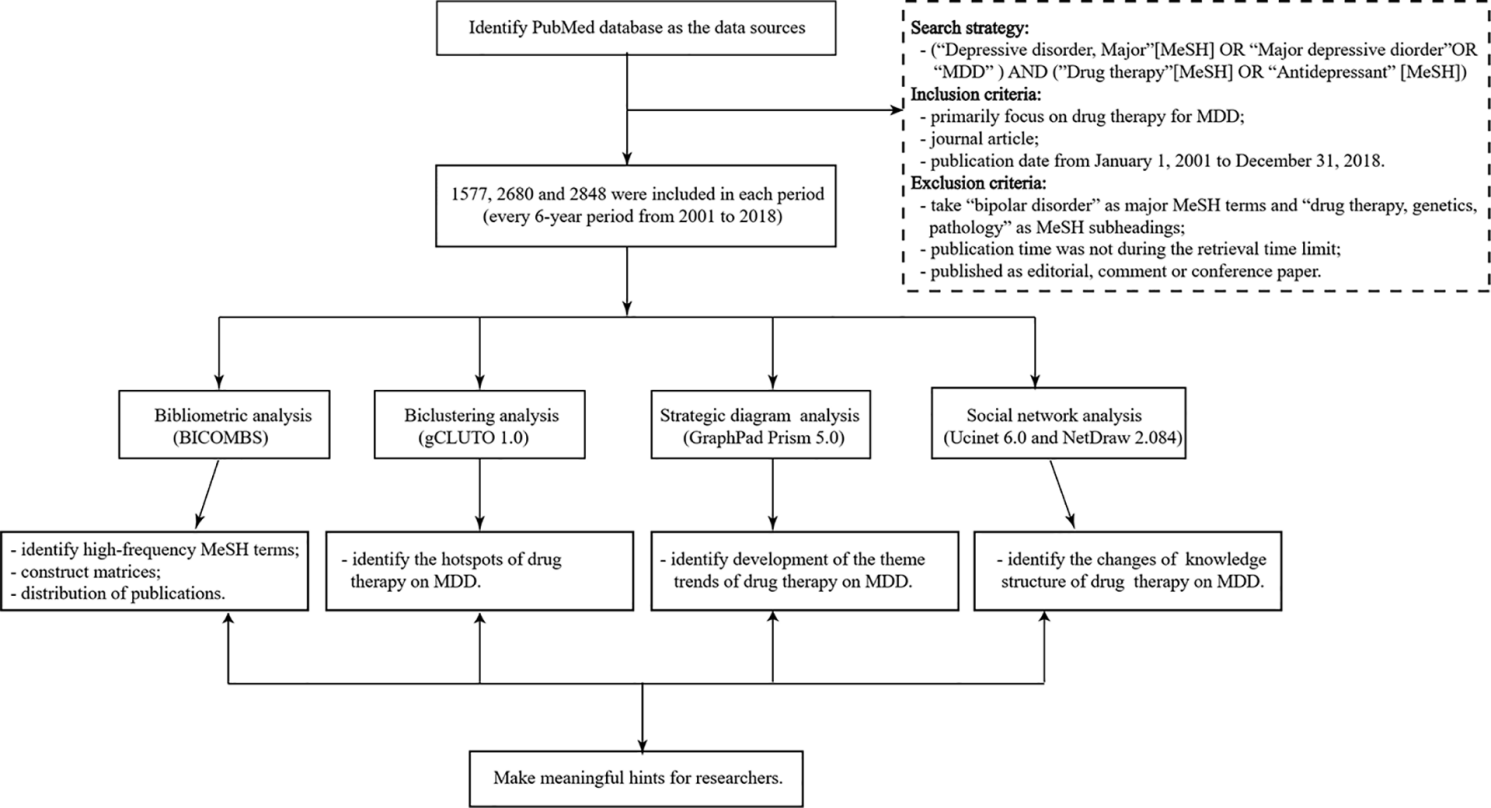
The flowchart of this bibliometric research. MDD, major depressive disorder; BICOMS, the bibliographic item co-occurrence matrix system. The contents in parentheses refer to software used to perform the targeted statistical analysis. Matrices constructed by BICOMBS include the term-source literature matrix and the term-term cooccurrence matrix.

The flowchart of this bibliometric research can be seen in **Figure 1**.

## Results

### Distribution Characteristics of Related Publications

**Figure 2:**
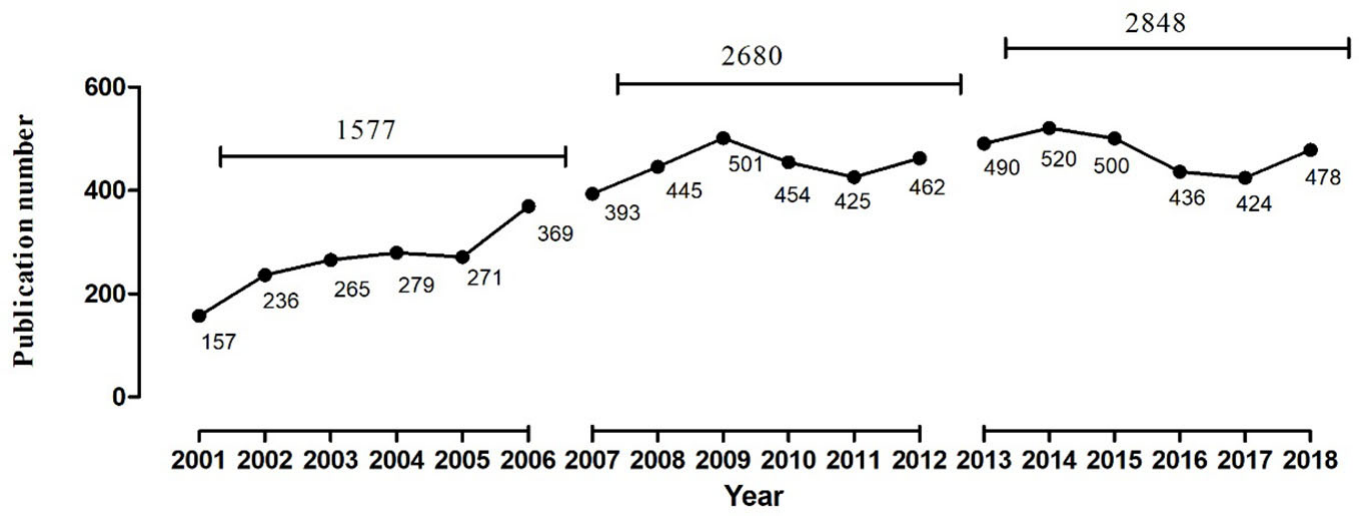
The number of publications of drug therapy studies on major depressive disorder in PubMed from 2001 to 2018.

As can be seen from **Figure 2**, the number of related articles that used MDD and drug therapy as the research subjects and had been published in PubMed was 1,577, 2,680, 2,848 from the three periods during 2001–2006, 2007–2012, and 2013–,2018, respectively, which showed a gradually increasing publication tendency. According to comparative statistical analysis by source of countries, journals, and the first author as the main statistical indices, we found that the total proportion of publications of the top five countries was 86.209%, 86.431%, and 84.027% during the three time-periods, respectively. The U.S., England, the Netherlands, and Germany were the top four countries in descending order of number of publications during each time-period (**Table 1**). Although France ranked fifth during 2001–2006, it was replaced by Ireland during the next two time-periods. Additionally, the proportion of publications in the U.S. has been trending downward, while in England and the Netherlands it has been increasing. Another important result was that *The Journal of Clinical Psychiatry* and *Journal of Affective Disorders* were first and second, respectively, in the number and proportion of publications during 2001–2006 and 2007–2012, but this order was reversed during 2013–2018. In addition, *Fava M* has been the author who contributed the most research papers to the research field of MDD and drug therapy during the three time-periods studied, although the number and proportion of his publications decreased during each successive time-period.

**Table 1:** Temporal distribution of publications of drug therapy studies on major depressive disorder in PubMed since the 21^st^ century.

Period	Rank	Country		Top journal		Author	
		Name	Publications, n (%)	Title	Publication, n (%)	Name	Number of papers, n (%)
2001-2006	1	United States	798 (46.831%)	*The Journal of clinical psychiatry*	170 (9.971%)	Fava M	90 (5.282%)
	2	England	419 (24.589%)	*Journal of affective disorders*	89 (5.220%)	Thase ME	52 (3.052%)
	3	Netherlands	124 (7.277%)	*Biological psychiatry*	64 (3.754%)	Rush AJ	51 (2.993%)
	4	Germany	81 (4.754%)	*Journal of clinical psychopharmacology*	53 (3.109%)	Nierenberg AA	48 (2.817%)
	5	France	47 (2.758%)	*The American journal of psychiatry*	43 (2.522%)	Trivedi MH	39 (2.289%)
	Total		1469 (86.209%)		419 (24.576%)		280 (16.433%)
2007-2012	1	United States	1235 (42.748%)	*The Journal of clinical psychiatry*	220 (7.613%)	Fava M	111 (3.842%)
	2	England	785 (27.172%)	*Journal of affective disorders*	217 (7.509%)	Thase ME	78 (2.700%)
	3	Netherlands	316 (10.938%)	*Depression and anxiety*	65 (2.250%)	Trivedi MH	77 (2.665%)
	4	Germany	90 (3.115%)	*International clinical psychopharmacology*	62 (2.145%)	Rush AJ	62 (2.146%)
	5	Ireland	71 (2.458%)	*Progress in neuro-psychopharmacology & biological psychiatry*	57 (1.972%)	Papakostas GI	56 (1.938%)
	Total		2497 (86.431%)		621 (21.489%)		384 (13.291%)
2013-2018	1	United States	1023 (34.294%)	*Journal of affective disorders*	285 (9.545%)	Fava M	60 (2.011%)
	2	England	942 (31.579%)	*The Journal of clinical psychiatry*	132 (4.421%)	McIntyre RS	59 (1.978%)
	3	Netherlands	443 (14.851%)	*Journal of psychiatric research*	72 (2.411%)	Thase ME	53 (1.777%)
	4	Germany	108 (3.621%)	*The international journal of neuropsychopharmacology*	60 (2.009%)	Trivedi MH	49 (1.643%)
	5	Ireland	80 (2.682%)	*Psychiatry research*	58 (1.942%)	Zarate CA	48 (1.609%)
	Total		2596 (84.027%)		607 (20.328%)		269 (9.018%)

### Research Hotspots and Theme Clusters of Drug Therapy Studies on MDD

In this study, 29, 35, and 36 high-frequency major MeSH terms/MeSH subheadings were extracted based on bibliographic statistical analysis from the periods during 2001–2006, 2007–2012, and 2013–2018, respectively. Further analysis demonstrated that the total percentages of their cumulative frequency were 43.6771%, 44.3149%, and 43.0717%, respectively, and that these extracted high-frequency major MeSH terms/MeSH subheadings could be identified as the research hotspots of drug therapy studies on MDD in every 6-year period from 2001 to 2018 (**Tables 2**–**4**).

**Table 2:** Distribution of the high-frequency major MeSH terms/MeSH subheadings of drug therapy studies on major depressive disorder in PubMed from 2001 to 2006.

Rank number	Major MeSH terms/ MeSH subheadings	Frequency	Percentage (%)	Cumulative percentage (%)
1	Depressive Disorder, Major / drug therapy	810	10.4949	10.4949
2	Antidepressive Agents / therapeutic use	554	7.1780	17.6730
3	Depressive Disorder, Major / therapy	205	2.6561	20.3291
4	Serotonin Uptake Inhibitors / therapeutic use	200	2.5913	22.9204
5	Antidepressive Agents, Second-Generation / therapeutic use	180	2.3322	25.2527
6	Depressive Disorder / drug therapy	156	2.0212	27.2739
7	Antidepressive Agents, Tricyclic / therapeutic use	100	1.2957	28.5696
8	Depressive Disorder, Major / diagnosis	98	1.2698	29.8393
9	Depressive Disorder, Major / psychology	86	1.1143	30.9536
10	Depressive Disorder, Major / epidemiology	75	0.9718	31.9254
11	Citalopram / therapeutic use	73	0.9458	32.8712
12	Fluoxetine / therapeutic use	72	0.9329	33.8041
13	Antidepressive Agents / adverse effects	68	0.8811	34.6852
14	Cyclohexanols / therapeutic use	64	0.8292	35.5144
15	Antidepressive Agents / administration & dosage	63	0.8163	36.3307
16	Paroxetine / therapeutic use	55	0.7126	37.0433
17	Sertraline / therapeutic use	49	0.6349	37.6782
18	Antidepressive Agents / pharmacology	49	0.6349	38.3130
19	Depressive Disorder, Major / metabolism	47	0.6090	38.9220
20	Depressive Disorder, Major / physiopathology	44	0.5701	39.4921
21	Antipsychotic Agents / therapeutic use	44	0.5701	40.0622
22	Depressive Disorder, Major / blood	38	0.4924	40.5545
23	Anxiety Disorders / drug therapy	38	0.4924	41.0469
24	Depressive Disorder, Major / complications	37	0.4794	41.5263
25	Depressive Disorder, Major / etiology	36	0.4664	41.9927
26	Thiophenes / therapeutic use	34	0.4405	42.4333
27	Depressive Disorder, Major / genetics	33	0.4276	42.8608
28	Mianserin / analogs & derivatives	33	0.4276	43.2884
29	Depressive Disorder / therapy	30	0.3887	43.6771

**Table 3:** Distribution of the high-frequency major MeSH terms/MeSH subheadings of drug therapy studies on major depressive disorder in PubMed from 2007 to 2012.

Rank number	Major MeSH terms/ MeSH subheadings	Frequency	Percentage (%)	Cumulative percentage (%)
1	Depressive Disorder, Major / drug therapy	1470	11.0543	11.0543
2	Antidepressive Agents / therapeutic use	1078	8.1065	19.1608
3	Depressive Disorder, Major / therapy	364	2.7373	21.8980
4	Antidepressive Agents, Second-Generation / therapeutic use	253	1.9025	23.8006
5	Serotonin Uptake Inhibitors / therapeutic use	207	1.5566	25.3572
6	Depressive Disorder, Major / psychology	174	1.3085	26.6657
7	Citalopram / therapeutic use	173	1.3009	27.9666
8	Depressive Disorder, Major / diagnosis	151	1.1355	29.1021
9	Depressive Disorder, Major / epidemiology	143	1.0753	30.1775
10	Depressive Disorder, Major / genetics	131	0.9851	31.1626
11	Antidepressive Agents / pharmacology	126	0.9475	32.1101
12	Cyclohexanols / therapeutic use	122	0.9174	33.0275
13	Antidepressive Agents / adverse effects	118	0.8874	33.9149
14	Depressive Disorder / drug therapy	110	0.8272	34.7421
15	Depressive Disorder, Major / physiopathology	109	0.8197	35.5617
16	Antipsychotic Agents / therapeutic use	105	0.7896	36.3513
17	Antidepressive Agents / administration & dosage	97	0.7294	37.0808
18	Thiophenes / therapeutic use	95	0.7144	37.7952
19	Fluoxetine / therapeutic use	86	0.6467	38.4419
20	Depressive Disorder, Major / blood	69	0.5189	38.9607
21	Piperazines / therapeutic use	62	0.4662	39.4270
22	Antidepressive Agents, Tricyclic / therapeutic use	60	0.4512	39.8782
23	Depressive Disorder, Major / complications	55	0.4136	40.2918
24	Sertraline / therapeutic use	54	0.4061	40.6978
25	Depressive Disorder, Major / metabolism	54	0.4061	41.1039
26	Mianserin / analogs & derivatives	51	0.3835	41.4874
27	Depression / drug therapy	51	0.3835	41.8710
28	Cognitive Behavioral Therapy	48	0.3610	42.2319
29	Serotonin Uptake Inhibitors / adverse effects	45	0.3384	42.5703
30	Depressive Disorder, Major / pathology	41	0.3083	42.8786
31	Anxiety Disorders / drug therapy	41	0.3083	43.1869
32	Cognitive Behavioral Therapy / methods	39	0.2933	43.4802
33	Paroxetine / therapeutic use	38	0.2858	43.7660
34	Antidepressive Agents, Second-Generation / adverse effects	37	0.2782	44.0442
35	Psychotherapy / methods	36	0.2707	44.3149

**Table 4:** Distribution of the high-frequency major MeSH terms/MeSH subheadings of drug therapy studies on major depressive disorder in PubMed from 2013 to 2018.

Rank number	Major MeSH terms/ MeSH subheadings	Frequency	Percentage (%)	Cumulative percentage (%)
1	Depressive Disorder, Major / drug therapy	1433	10.1986	10.1986
2	Antidepressive Agents / therapeutic use	1206	8.5830	18.7816
3	Depressive Disorder, Major / therapy	368	2.6190	21.4006
4	Antidepressive Agents / pharmacology	242	1.7223	23.1229
5	Depressive Disorder, Major / psychology	193	1.3736	24.4965
6	Depressive Disorder, Major / diagnosis	157	1.1174	25.6138
7	Depression / drug therapy	154	1.0960	26.7098
8	Serotonin Uptake Inhibitors / therapeutic use	147	1.0462	27.7560
9	Antidepressive Agents, Second-Generation / therapeutic use	144	1.0248	28.7809
10	Citalopram / therapeutic use	136	0.9679	29.7488
11	Antidepressive Agents / administration & dosage	133	0.9466	30.6953
12	Depressive Disorder, Major / genetics	132	0.9394	31.6348
13	Depressive Disorder, Treatment-Resistant / drug therapy	128	0.9110	32.5457
14	Depressive Disorder, Major / physiopathology	118	0.8398	33.3855
15	Antidepressive Agents / adverse effects	109	0.7757	34.1613
16	Depressive Disorder, Major / metabolism	91	0.6476	34.8089
17	Antipsychotic Agents / therapeutic use	89	0.6334	35.4423
18	Depressive Disorder, Major / blood	81	0.5765	36.0188
19	Depressive Disorder, Major / epidemiology	78	0.5551	36.5739
20	Piperazines / therapeutic use	73	0.5195	37.0934
21	Depressive Disorder / drug therapy	72	0.5124	37.6059
22	Depressive Disorder, Major / complications	70	0.4982	38.1040
23	Ketamine / therapeutic use	68	0.4840	38.5880
24	Brain / drug effects	60	0.4270	39.0150
25	Cognitive Behavioral Therapy / methods	59	0.4199	39.4349
26	Sertraline / therapeutic use	57	0.4057	39.8406
27	Depressive Disorder, Major / pathology	51	0.3630	40.2035
28	Ketamine / pharmacology	51	0.3630	40.5665
29	Fluoxetine / therapeutic use	49	0.3487	40.9152
30	Outcome Assessment (Health Care)	48	0.3416	41.2569
31	Sulfides / therapeutic use	46	0.3274	41.5842
32	Depressive Disorder, Treatment-Resistant / therapy	46	0.3274	41.9116
33	Depression / therapy	44	0.3131	42.2248
34	Cyclohexanols / therapeutic use	43	0.3060	42.5308
35	Psychotherapy / methods	40	0.2847	42.8155
36	Suicidal Ideation	36	0.2562	43.0717

**Figure 3:**
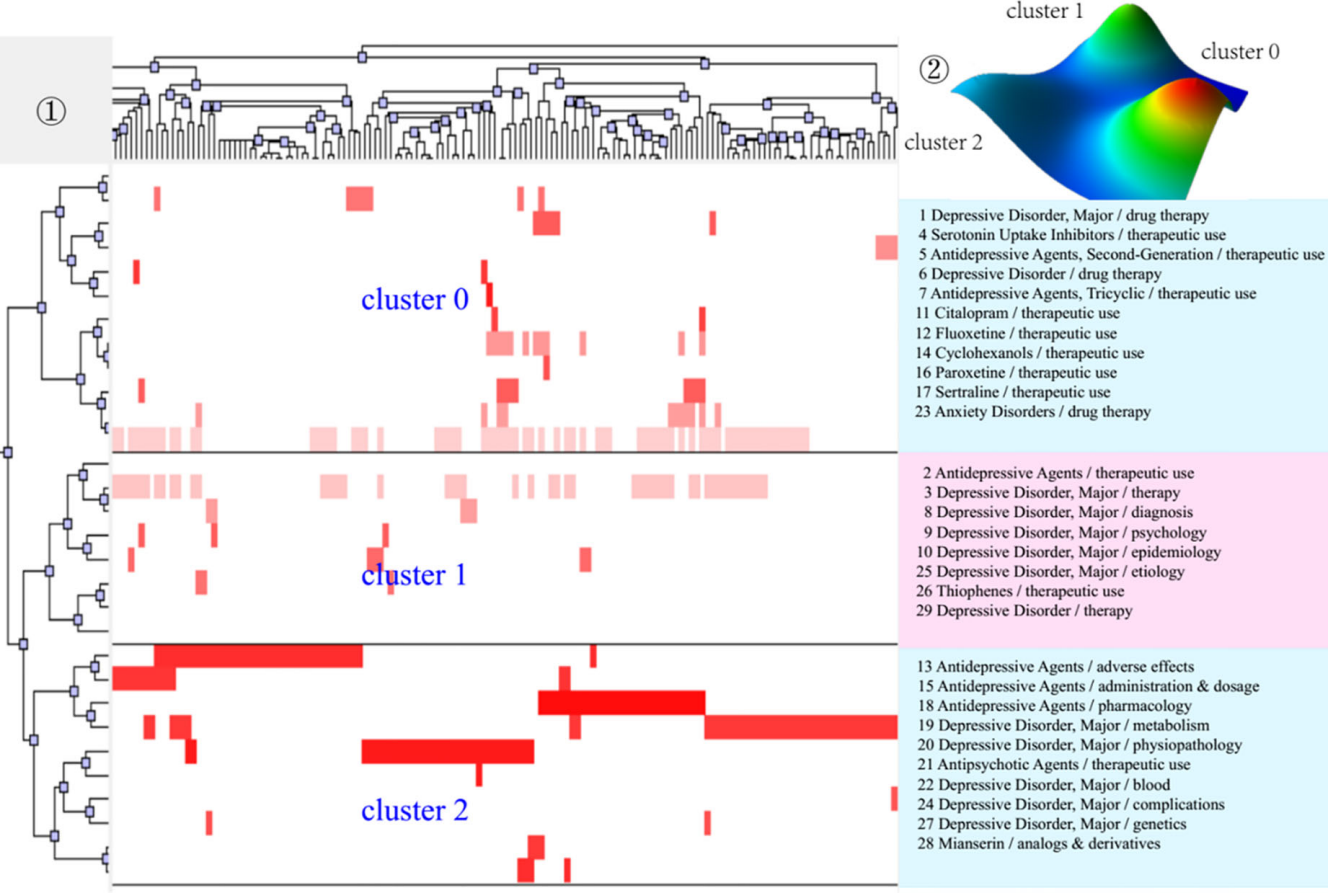
Bi-clustering analysis of 29 high-frequency MeSH terms/MeSH subheadings and literatures of drug therapy studies on major depressive disorder in 2001–2006. ①Matrix visualization of bi-clustering of 29 high-frequency major MeSH terms/MeSH subheadings and PubMed unique identifiers of literatures. ②Mountain visualization of bi-clustering of 29 high-frequency major MeSH terms/MeSH subheadings and literatures.

**Figure 4:**
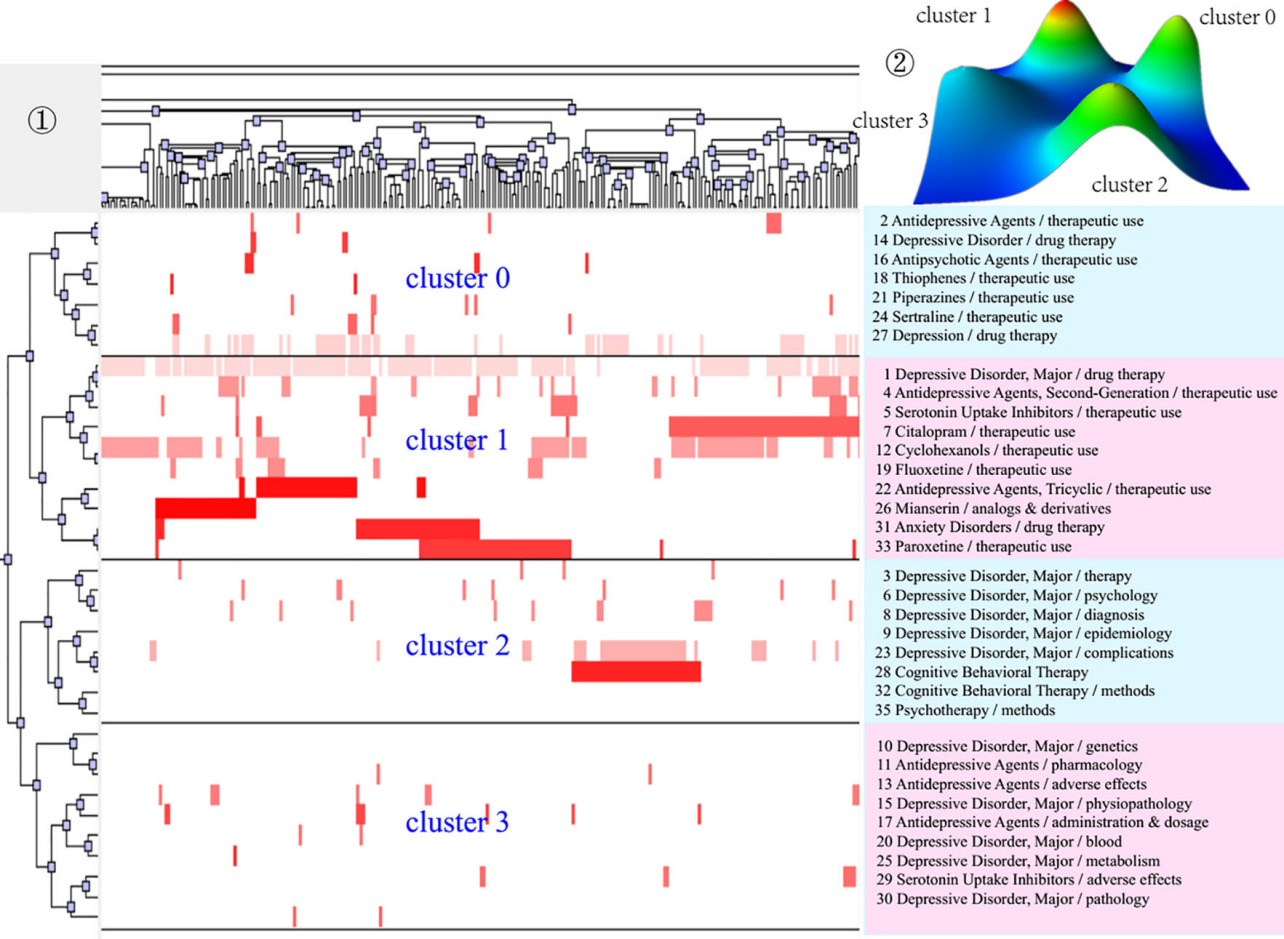
Bi-clustering analysis of 35 high-frequency MeSH terms/MeSH subheadings and literatures of drug therapy studies on major depressive disorder in 2007–2012. ①Matrix visualization of bi-clustering of 35 high-frequency major MeSH terms/MeSH subheadings and PubMed unique identifiers of literatures. ②Mountain visualization of bi-clustering of 35 high-frequency major MeSH terms/MeSH subheadings and literatures.

**Figure 5:**
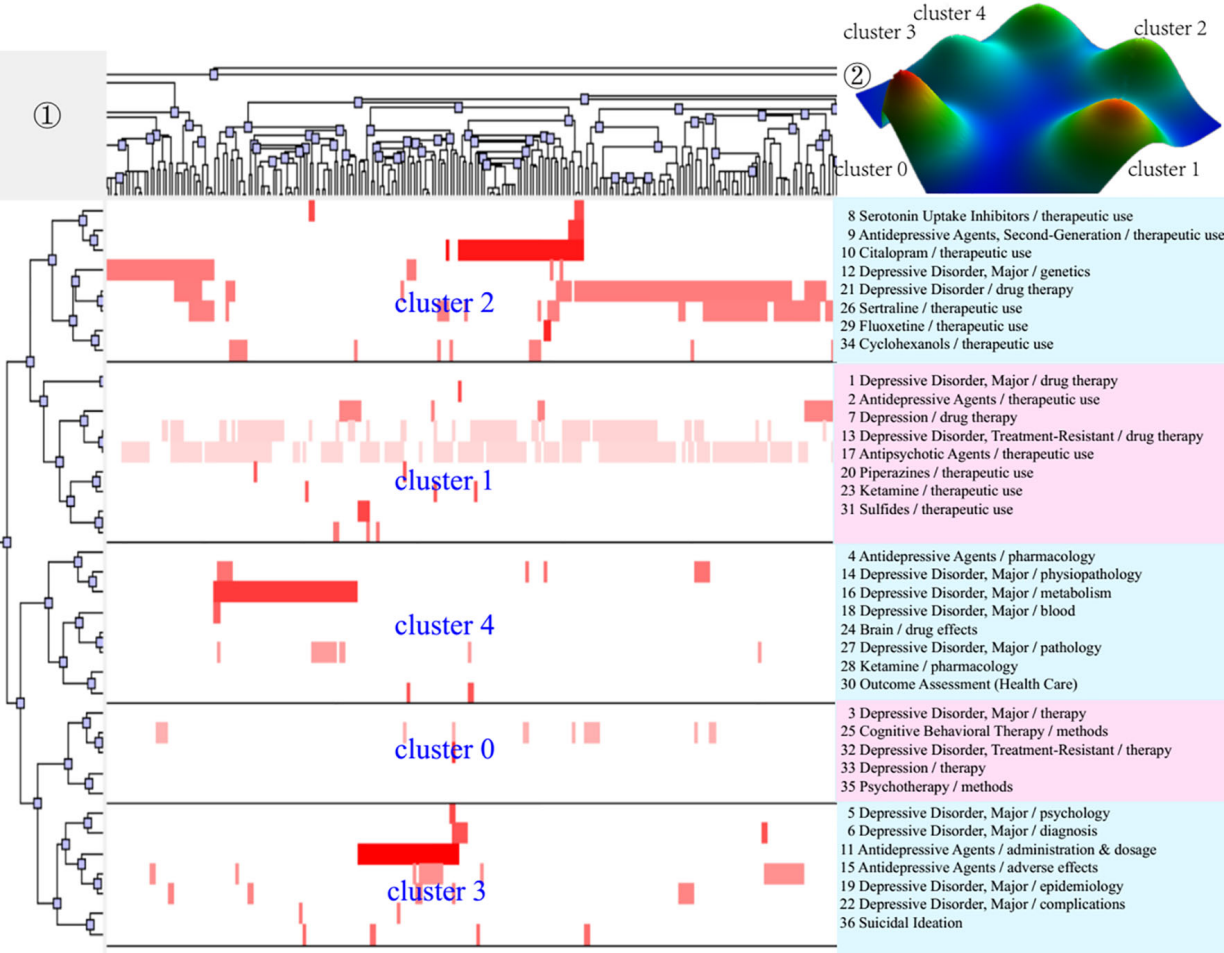
Bi-clustering analysis of 36 high-frequency MeSH terms/MeSH subheadings and literatures of drug therapy studies on major depressive disorder in 2013–2018. ①Matrix visualization of bi-clustering of 36 high-frequency major MeSH terms/MeSH subheadings and PubMed unique identifiers of literatures. ②Mountain visualization of bi-clustering of 36 high-frequency major MeSH terms/MeSH subheadings and literatures.

After completing bi-clustering analysis, three, four, and five theme clusters were obtained based on actual clustering effects and researchers’ professional knowledge in the research field of MDD and drug therapy, and a visual matrix and visual mountain of three different periods were generated (**Figures 3**–**5**).

As for matrix visualization, the color represents values in the original data matrix of term-source literature. Specifically, white stands for near-zero values, while deepening red is indicative of larger values. The rows in the matrix are rearranged so that the rows and columns of the theme cluster are placed together. Black horizontal lines separate these generated clusters in each period. Besides, we also annotated the extracted high-frequency major MeSH terms/MeSH subheadings in each theme cluster on the right side of the matrix (**Figures 3**–**5**), with the serial number before MeSH terms having the same meaning as in **Tables 2**–**4**.

Within mountain visualization, the shape of each mountain is actually a Gaussian curve, which represents a rough estimate of the distribution of data within each theme cluster. The height and volume of the mountain are proportional to the internal similarity within the cluster, and the number of objects contained in each theme cluster, respectively. Several Gaussian curves are then arranged and superimposed on the basis of bi-clustering statistical results and thus form a mountain visualization. The color of the mountain is proportional to the standard deviation (SD) within each theme cluster. Moreover, only the color of the summit is relevant, where red represents low SD, and blue represents high. The peak of cluster 0 during 2001–2006, cluster 1 during 2007–2012, and cluster 1 and cluster 3 during 2013–2018 are red, indicating that the SD of similarity within the theme clusters is the smallest; that is, the distribution is concentrated.

We further combined contents of the high-frequency major MeSH terms/MeSH subheadings contained in each cluster, the extracted significant representative literature (seen column 5 in **Table 6**), and researchers’ professional knowledge in the field of drug therapy studies on MDD to interpret meaning and connation of each theme cluster involved during the three time-periods from 2001 to 2018.

### Theme Trends of Drug Therapy Studies on MDD

**Figure 6:**
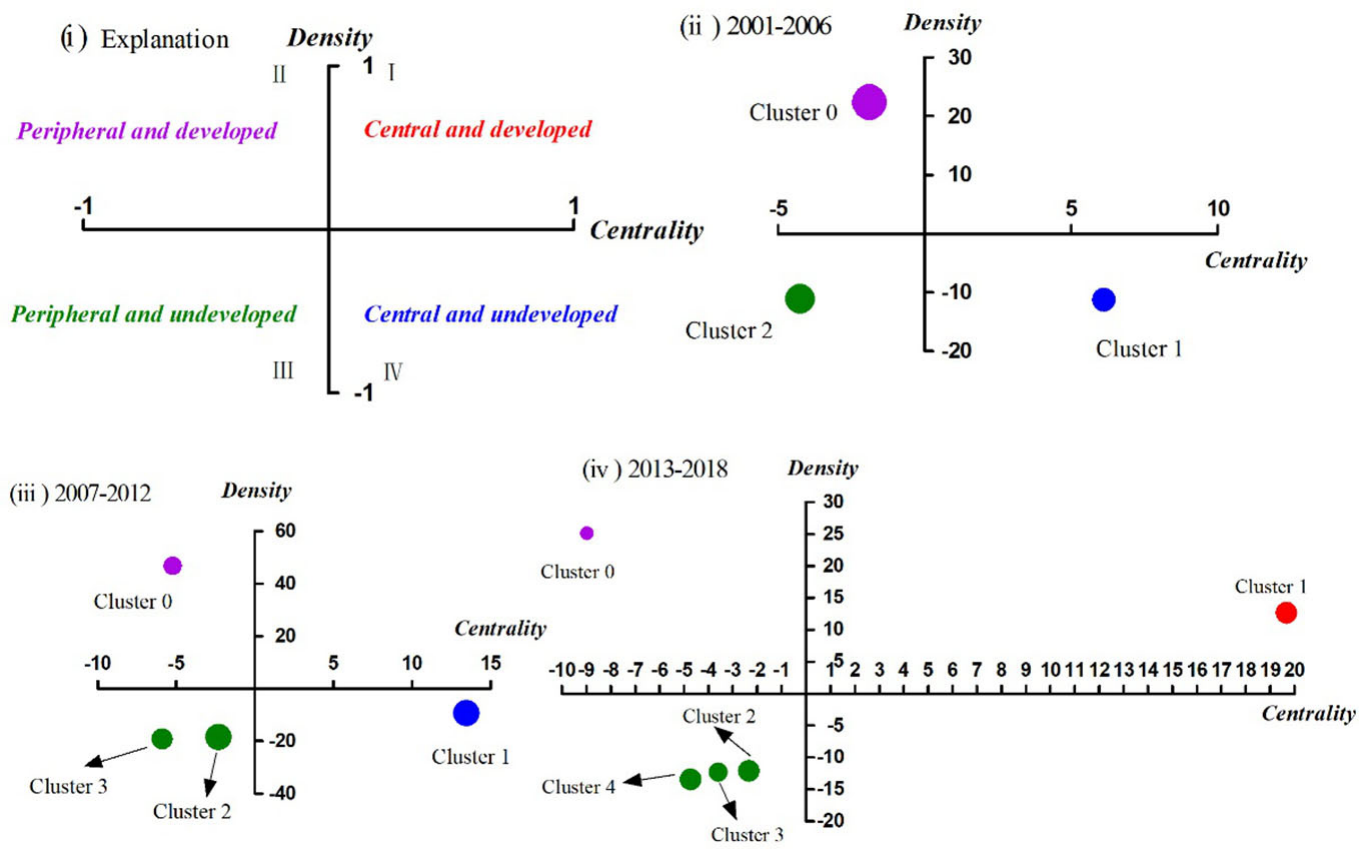
Strategic diagrams for drug therapy studies on major depressive disorder in three different periods. (i) Explanation of the strategic diagram. (ii) Strategic diagram in 2001–2006. (iii) Strategic diagram in 2007–2012. (iv) Strategic diagram in 2013–2018. Clusters in each strategic diagram refer to the bi-clustering results presented in **Table 7**. The size of each single node is proportional to the number of high-frequency major MeSH terms/MeSH subheadings involved in each cluster (**Table 6**).

By defining the baseline (mean and median), the four quadrants of the strategic diagram can be obtained, where each quadrant reflects the development of the theme cluster (49). As shown in **Figure 6(i)**, clusters in quadrant I have the strongest centrality and density. The clusters are also the core themes of their research field, are closely related to each other, and have been developed due to researchers’ extensive attention. Clusters in quadrant II have higher density, but lower centrality, indicating that the hotspots/MeSH terms within the clusters are closely related, but that the relationship with other hotspots/MeSH terms outside the cluster is relatively distant. In quadrant III, the centrality and density of the clusters are both the weakest, representing that they are neither closely related within clusters nor with other external clusters, and they display immaturity in development. Clusters with stronger centrality and lower density in quadrant IV indicate loose internal structure and immature development. However, contents in quadrant IV are closely related to other clusters and topics in the cluster have been actively pursued in the research field, which deserves attention.

Between 2001–2006, cluster 0 in quadrant II represented the application and therapeutic effect of different types of antidepressant agents, which was considered as peripheral but developed content. Cluster 1 in quadrant IV represented analysis of influencing factors of the therapeutic effect and clinical outcome (e.g., adverse reaction) of antidepressant drugs, which were central, but undeveloped themes. The contents of cluster 2 referred to research on the functional mechanism of antidepressants in pathophysiology, neuroendocrinology, and neurobiochemistry, whose coordinate was in quadrant III, indicating that the related content was considered to be either emerging or in decline, namely on the edge of the research field during that period of time.

Compared with the results of the first period, the causes of adverse reactions of antidepressant agents, which were part of cluster 3, had transferred from quadrant IV in 2001–2006 to quadrant III in 2007–2012. Additionally, other parts of cluster 3 were predictive studies on the effectiveness of antidepressant treatment based on brain imaging, which was a newly developed theme. Cluster 0, located in quadrant II, mainly focused on comparative analysis of the clinical treatment effect and tolerability of antidepressant agents (mainly SSRIs and SNRIs) combined with antipsychotics in patients with depressive disorder, while cluster 1 in quadrant IV focused on analysis of the therapeutic effect of different antidepressant agents (mainly SSRIs) on patients with major depressive disorder, as well as analysis of the effect of different antidepressants on the treatment of patients with MDD comorbid with anxiety. Contents of cluster 2, located in quadrant III, was part of the same as cluster 1 during 2001–2006, but research into the effects of psychotherapy interventions were added to it.

During 2013–2018, cluster 0, which focused on analysis of the therapeutic effect and influencing factors of patients with depressive disorder after receiving psychotherapy alone or combined with drug therapy, was part of the same theme content as cluster 2 (quadrant III) during 2007–2012, but transferred to quadrant II with higher density but lower centrality. Cluster 1, situated in quadrant I, identified as developed and core themes in the past 6 years, represented evaluation of the therapeutic effect of new antidepressants, as well as clinical effects of antipsychotic drugs combined with antidepressant agents. By comparison, we found that the second part of cluster 1, which was the same as cluster 0 (quadrant II) during 2007–2012, whereby, the theme content gradually moved from the peripheral to the core. Moreover, clusters 2, 3, and 4 were situated in quadrant III, and mainly focused on comparative analysis of clinical treatment effects of new antidepressant agents and other SSRIs with genetic/biomarkers that predict the effectiveness of antidepressant therapy; the effects of antidepressant treatment on residual depressive symptoms; the mechanism of action; and clinical therapeutic effects of antidepressant agents.

In brief, these three strategic diagrams clearly revealed the current situation and development tendency of each theme cluster of drug therapy studies on MDD during the three different time-periods.

### Knowledge Structure of Drug Therapy Studies on MDD

**Figure 7:**
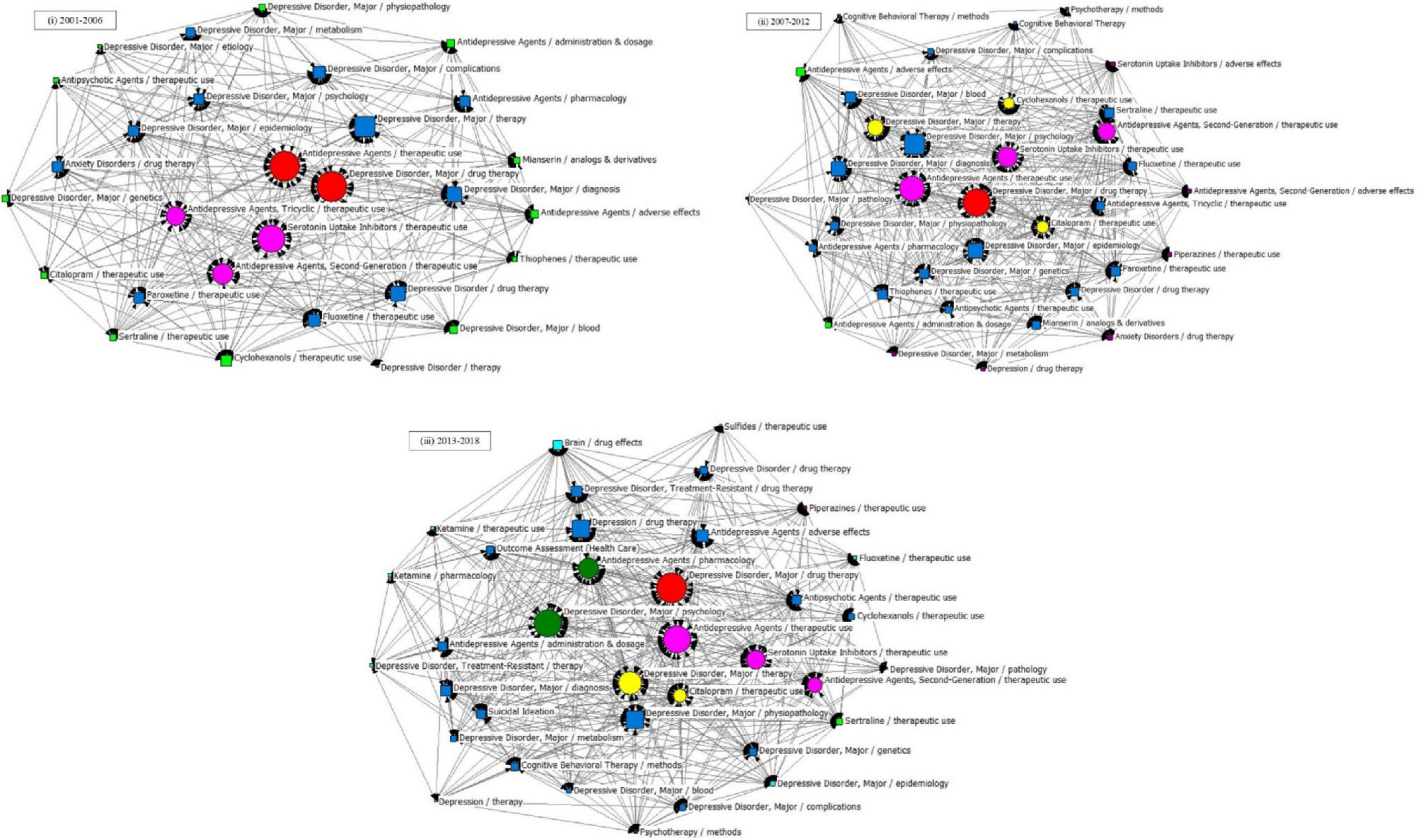
SNA for high-frequency major MeSH terms/MeSH subheadings applied to drug therapy studies on major depressive disorder. (i) SNA for 29 high-frequency major MeSH terms/MeSH subheadings in 2001–2006. (ii) SNA for 35 high-frequency major MeSH terms/MeSH subheadings in 2007–2012. (iii) SNA for 36 high-frequency major MeSH terms/MeSH subheadings in 2013–2018. The size of the nodes and the thickness of the lines within the networks represent centrality of MeSH terms and the co-occurrence frequency of MeSH terms pairs, respectively. Red icons (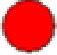): Node with the highest betweenness centrality. Circle icons: Nodes highly co-occurred with other nodes since the period of 2001–2006 (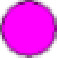), 2007–2012 (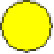) and 2013–2018 (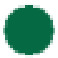), respectively (**Figure 7** (i)–(iii)). Box icons: Emerging nodes in 2001–2006 (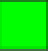), 2007–2012 (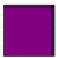) and 2013–2018(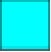), respectively.

Results of the SNAs of the three periods are presented in **Figure 7**, and the main statistical parameters, such as degree, betweenness, and closeness centrality, were employed to analyze the knowledge structure of drug therapy studies on MDD between 2001 and 2018 (**Tables 5** and **6**).

**Table 5:** Descriptive statistics for centrality measures of drug therapy studies on major depressive disorder since the 21^st^ century.

Period	Density	Degree			Betweenness			Closeness		
		Min	Max	$\bar{x}$±S	Min	Max	$\bar{x}$±S	Min	Max	$\bar{x}$±S
2001-2006	7.096±21.748	23.000	1321	198.690±268.690	0.000	13.055	4.552±3.444	58.333	100	76.702±10.120
2007-2012	8.886±35.781	32.000	2471	302.114±480.325	0.059	17.598	5.486±4.190	59.649	100	76.886±10.056
2013-2018	8.357±35.781	52.000	2378	292.500±478.819	0.484	18.869	5.917±4.987	60.345	100	76.099±10.548

**Table 6:** Descriptive statistics for centrality measure of drug therapy studies on major depressive disorder since the 21^st^ century.

Period	Rank of MeSH terms	High-frequency MeSH terms/MeSH subheadings	Centrality			Rank of MeSH terms	High-frequency MeSH terms/MeSH subheadings	Centrality		
			Degree	Betweenness	Closeness			Degree	Betweenness	Closeness
2001-2006	1	Depressive Disorder, Major / drug therapy	1321.000	13.055	100.000	23	Anxiety Disorders / drug therapy	87.000	4.037	75.676
	2	Antidepressive Agents / therapeutic use	836.000	13.055	100.000	15	Antidepressive Agents / administration & dosage	84.000	2.673	68.293
	4	Serotonin Uptake Inhibitors / therapeutic use	499.000	11.642	96.552	26	Thiophenes / therapeutic use	84.000	1.349	66.667
	5	Antidepressive Agents, Second-Generation / therapeutic use	426.000	8.323	87.500	13	Antidepressive Agents / adverse effects	79.000	2.773	70.000
	7	Antidepressive Agents, Tricyclic / therapeutic use	224.000	7.926	87.500	18	Antidepressive Agents / pharmacology	70.000	3.980	71.795
	3	Depressive Disorder, Major / therapy	194.000	8.658	87.000	21	Antipsychotic Agents / therapeutic use	67.000	1.399	66.667
	6	Depressive Disorder / drug therapy	192.000	5.687	77.778	27	Depressive Disorder, Major / genetics	67.000	1.908	68.293
	11	Citalopram / therapeutic use	189.000	2.101	71.795	24	Depressive Disorder, Major / complications	66.000	4.847	77.778
	12	Fluoxetine / therapeutic use	184.000	4.508	80.000	28	Mianserin / analogs & derivatives	63.000	1.966	68.293
	14	Cyclohexanols / therapeutic use	183.000	4.236	77.778	19	Depressive Disorder, Major / metabolism	62.000	3.247	71.795
	16	Paroxetine / therapeutic use	146.000	3.799	80.000	25	Depressive Disorder, Major / etiology	55.000	0.903	65.116
	9	Depressive Disorder, Major / psychology	119.000	3.719	77.778	20	Depressive Disorder, Major / physiopathology	55.000	2.211	70.000
	17	Sertraline / therapeutic use	118.000	1.825	73.684	22	Depressive Disorder, Major / blood	51.000	2.393	70.000
	8	Depressive Disorder, Major / diagnosis	112.000	6.155	80.000	29	Depressive Disorder / therapy	23.000	0.000	58.333
	10	Depressive Disorder, Major / epidemiology	106.000	3.625	77.778	–	–	–	–	–
2007-2012	1	Depressive Disorder, Major / drug therapy	2471.000	17.598	100.000	22	Antidepressive Agents, Tricyclic / therapeutic use	146.000	4.136	79.070
	2	Antidepressive Agents / therapeutic use	1815.00	15.461	97.143	11	Antidepressive Agents / pharmacology	142.000	3.964	72.340
	4	Antidepressive Agents, Second-Generation / therapeutic use	623.000	10.759	89.474	15	Depressive Disorder, Major / physiopathology	140.000	4.513	75.556
	5	Serotonin Uptake Inhibitors / therapeutic use	562.000	12.658	94.444	26	Mianserin / analogs & derivatives	125.000	4.107	75.556
	7	Citalopram / therapeutic use	455.000	7.196	87.179	17	Antidepressive Agents / administration & dosage	118.000	3.062	68.000
	3	Depressive Disorder, Major / therapy	412.000	10.015	85.000	20	Depressive Disorder, Major / blood	109.000	6.497	80.952
	12	Cyclohexanols / therapeutic use	349.000	5.931	82.927	33	Paroxetine / therapeutic use	108.000	5.242	77.273
	6	Depressive Disorder, Major / psychology	289.000	12.019	89.474	28	Cognitive Behavioral Therapy	108.000	1.291	66.667
	10	Depressive Disorder, Major / genetics	244.000	5.620	79.070	31	Anxiety Disorders / drug therapy	99.000	2.435	69.388
	18	Thiophenes / therapeutic use	242.000	5.242	79.070	27	Depression / drug therapy	84.000	1.963	69.388
	19	Fluoxetine / therapeutic use	241.000	4.217	77.273	29	Serotonin Uptake Inhibitors / adverse effects	81.000	2.161	69.388
	9	Depressive Disorder, Major / epidemiology	237.000	9.178	87.179	32	Cognitive Behavioral Therapy / methods	70.000	0.558	60.714
	16	Antipsychotic Agents / therapeutic use	208.000	3.594	75.556	23	Depressive Disorder, Major / complications	68.000	3.094	69.388
	8	Depressive Disorder, Major / diagnosis	196.000	8.957	85.000	35	Psychotherapy / methods	67.000	0.555	60.714
	13	Antidepressive Agents / adverse effects	166.000	4.314	73.913	25	Depressive Disorder, Major / metabolism	53.000	1.986	66.667
	14	Depressive Disorder / drug therapy	166.000	4.713	75.556	34	Antidepressive Agents, Second-Generation / adverse effects	49.000	1.746	65.385
	21	Piperazines / therapeutic use	150.000	1.536	69.388	30	Depressive Disorder, Major / pathology	32.000	0.059	59.649
	24	Sertraline / therapeutic use	149.000	5.628	77.273	–	–	–	–	–
2013-2018	1	Depressive Disorder, Major / drug therapy	2378.000	18.869	100.000	23	Ketamine / therapeutic use	149.000	1.495	64.815
	2	Antidepressive Agents / therapeutic use	2059.000	17.041	97.222	31	Sulfides / therapeutic use	147.000	0.484	61.404
	3	Depressive Disorder, Major / therapy	510.000	14.435	94.595	24	Brain / drug effects	138.000	4.971	76.087
	8	Serotonin Uptake Inhibitors / therapeutic use	361.000	11.084	87.500	25	Cognitive Behavioral Therapy / methods	122.000	4.199	72.917
	10	Citalopram / therapeutic use	349.000	8.026	85.366	16	Depressive Disorder, Major / metabolism	121.000	2.995	71.429
	4	Antidepressive Agents / pharmacology	342.000	12.020	89.744	29	Fluoxetine / therapeutic use	120.000	1.198	66.038
	5	Depressive Disorder, Major / psychology	324.000	17.486	97.222	18	Depressive Disorder, Major / blood	114.000	1.902	68.627
	9	Antidepressive Agents, Second-Generation / therapeutic use	315.000	8.362	83.333	34	Cyclohexanols / therapeutic use	112.000	2.797	70.000
	13	Depressive Disorder, Treatment-Resistant / drug therapy	272.000	5.951	77.778	30	Outcome Assessment (Health Care)	107.000	4.498	71.429
	6	Depressive Disorder, Major / diagnosis	265.000	6.067	81.395	21	Depressive Disorder / drug therapy	103.000	3.917	68.627
	7	Depression / drug therapy	243.000	10.778	83.333	19	Depressive Disorder, Major / epidemiology	94.000	2.334	71.429
	12	Depressive Disorder, Major / genetics	212.000	4.311	72.917	22	Depressive Disorder, Major / complications	91.000	3.235	71.429
	20	Piperazines / therapeutic use	212.000	1.347	66.038	28	Ketamine / pharmacology	87.000	1.778	66.038
	14	Depressive Disorder, Major / physiopathology	189.000	10.675	85.366	36	Suicidal Ideation	83.000	5.130	79.545
	11	Antidepressive Agents / administration & dosage	186.000	5.262	74.468	35	Psychotherapy / methods	79.000	1.817	67.308
	17	Antipsychotic Agents / therapeutic use	165.000	4.741	76.087	32	Depressive Disorder, Treatment-Resistant / therapy	66.000	1.790	63.636
	15	Antidepressive Agents / adverse effects	156.000	7.341	79.545	27	Depressive Disorder, Major / pathology	55.000	0.661	63.636
	26	Sertraline / therapeutic use	152.000	3.400	72.917	33	Depression / therapy	52.000	0.603	60.345

“Rank of MeSH terms” represents the number of high-frequency major MeSH terms/MeSH subheadings in each period as shown in **Tables 2**–**4**.

**Table 7:** Cluster interpretation of high-frequency major MeSH terms/MeSH subheadings of drug therapy studies on major depressive disorder in PubMed since the 21^st^ century.

Period	Cluster	Content and interpretation of the cluster	Rank of MeSH terms	Representative literatures (PMID)
2001-2006	Cluster 0	Application and therapeutic effect of different types of antidepressant agents (TCAs, SSRIs, etc.)	1, 4, 5, 6, 7, 11, 12, 14, 16, 17, 23	12490825, 15780704, 12027788, 16503815
	Cluster 1	Analysis of influencing factors of the therapeutic effect and clinical outcome (including therapeutic effect and adverse reaction) of antidepressant agents	2, 3, 8, 9, 10, 25, 26, 29	15679210, 12119755, 11497387, 16035056, 14653433, 16356553
	Cluster 2	Research on the functional mechanism of antidepressants in pathophysiology, neuroendocrinology, and neuro-biochemistry	13, 15, 18, 19, 20, 21, 22, 24, 27, 28	12478878, 14658930, 11274650, 15774440, 16388723
2007-2012	Cluster 0	Comparative analysis on the clinical treatment effect and tolerability of antidepressant agents (mainly SSRIs and SNRIs) combined with antipsychotics in patients with depressive disorder	2, 14, 16, 18, 21, 24, 27	22813840, 19210951, 17340654, 18164528
	Cluster 1	Analysis of the therapeutic effect of different antidepressant agents (mainly SSRI) on patients with major depressive disorder	1, 4, 5, 7, 12, 19, 22, 26, 31, 33	19345072, 19453203, 18299900
	Cluster 2	Analysis of the therapeutic effect and influencing factors of patients with depressive disorder after receiving psychotherapy (e.g., cognitive therapy, interpersonal psychotherapy) alone, or combined with drug therapy	3, 6, 8, 9, 23, 28, 32, 35	19798756, 22985486, 22700618, 19031487
	Cluster 3	1. Explore the causes of adverse reactions of antidepressant agents 2. Predictive studies on effectiveness of antidepressant treatment based on brain imaging	10, 11, 13, 15, 17, 20, 25, 29, 30, 34	17414739, 21824458, 20393460, 21546222
2013-2018	Cluster 0	1. Analysis on the therapeutic effect and influencing factors of patients with depressive disorder after receiving cognitive therapy alone, interpersonal psychotherapy, or combined with drug therapy 2. Analysis of the clinical therapeutic effect and cost-effectiveness of different psychotherapy methods	3, 25, 32, 33, 35	27236335, 24060588, 27461440, 25093396, 26348032
	Cluster 1	1. Evaluation of the mechanism of action and therapeutic effect of new antidepressants 2. Clinical effect of antipsychotic drugs combined with antidepressant agents	1, 2, 7, 13, 17, 20, 23, 31	25087600, 24676550, 27807822, 24045606, 23442899
	Cluster 2	1. Analysis of clinical treatment effect of the new antidepressant agents and other SSRIs 2. Study on genetic/biomarkers that influence the response and efficacy of antidepressant treatment	8, 9, 10, 12, 21, 26, 29, 34	23472671, 25815419, 24679400, 26234589
	Cluster 3	Effects of antidepressant treatment on residual symptoms of depressed patients and improvement methods	5, 6, 11, 15, 19, 22, 36	26415692, 27500820, 28445632
	Cluster 4	1. Analysis and research on the mechanism of action and clinical therapeutic effect of antidepressant agents 2. Early identification and prevention of suicide attempts and other risky behaviors of depressed patients	4, 14, 16, 18, 24, 27, 28, 30	26211972, 23271325, 28458140, 23442899

“Rank of MeSH terms” represents the number of high-frequency major MeSH terms/MeSH subheadings in each period as shown in **Tables 2**–**4**.

In the first period, during 2001–2006, five MeSH terms (the red and fuchsia circle nodes in **Figure 7i**), including “depressive disorder, major/drug therapy”, “antidepressive agents/therapeutic use”, “serotonin uptake inhibitors/therapeutic use”, “antidepressive agents, second-generation/therapeutic use”, “antidepressive agents, and tricyclic/therapeutic use”, were shown to have high degree centrality, with values greater than the mean value of 198.690. Among the above five MeSH terms, “depressive disorder, major/drug therapy” had the highest degree centrality at 1312.000 (**Table 6** and **Figure 7**). In addition, given that the size of a node of an SNA is measured by its betweenness centrality, we found that the MeSH terms “depressive disorder, major/drug therapy”, and “antidepressive agents/therapeutic use” displayed the highest same value of betweenness centrality of 13.055 and the highest same value of closeness centrality of 100.000 (**Table 6**). The results indicated that the above terms not only played the most significant mediating role, but also had a tight connection with other nodes in the SNA during 2001–2006. Meanwhile, as shown in **Table 5**, another seven MeSH terms, including “serotonin uptake inhibitors/therapeutic use”, “antidepressive agents, second-generation/therapeutic use”, “antidepressive agents, tricyclic/therapeutic use”, “depressive disorder, major/therapy”, “depressive disorder/drug therapy”, “depressive disorder, major/diagnosis”, and “depressive disorder, major/complications” displayed higher betweenness centrality whose values of betweenness degree were greater than the mean value of 4.552 (**Table 5**). Furthermore, 13 MeSH terms (lime square nodes in **Figure 7i**), such as “depressive disorder, major/physiopathology”, “antipsychotic agents/therapeutic use”, “depressive disorder, major/etiology”, “depressive disorder, major/genetics”, “citalopram/therapeutic use”, “cyclohexanols/therapeutic use”, “sertraline/therapeutic use”, “depressive disorder/therapy”, “depressive disorder, major/blood”, “antidepressive agents/adverse effects”, “thiophenes/therapeutic use”, “antidepressive agents/administration & dosage”, and “antidepressive agents/pharmacology” were emerging hotspots, which were located on the edge of the SNA during 2001–2006.

As in the first time-period, the MeSH term “depressive disorder, major/drug therapy” still had the highest value of degree centrality (2471.000, **Table 6** and **Figure 7ii**) during 2007–2012. Expect for the four MeSH terms (the red and fuchsia red nodes in **Figure 7ii**) with high values of degree centrality in 2001–2006, another three MeSH terms (yellow circle nodes in **Figure 7ii**) “citalopram/therapeutic use”, “depressive disorder, major/therapy”, and “cyclohexanols/therapeutic use” displayed higher degree centrality, with values greater than the mean value of 302.114 (**Table 5**). Whereas, 13 MeSH terms, including “depressive disorder, major/drug therapy”, “antidepressive agents/therapeutic use”, “antidepressive agents, second-generation/therapeutic use”, “serotonin uptake inhibitors/therapeutic use”, “citalopram/therapeutic use”, “depressive disorder, major/therapy”, “cyclohexanols/therapeutic use”, “depressive disorder, major/psychology”, “depressive disorder, major/genetics”, “depressive disorder, major/epidemiology”, “depressive disorder, major/diagnosis”, “sertraline/therapeutic use”, as well as “depressive disorder, major/blood”, displayed higher betweenness centrality (greater than the mean value of 5.486, **Table 5**). In addition, the newly nine emerging hotspots (purple square nodes in **Figure 7ii**) were located on the edge of the SNA during 2007–2012, including “antidepressive agents, second-generation/adverse effects”, “serotonin uptake inhibitors/adverse effects”, “depressive disorder, major/metabolism”, “depressive disorder, major/pathology”, “cognitive behavioral therapy/methods”, “depression/drug therapy”, “piperazines/therapeutic use”, “anxiety disorders/drug therapy”, and “psychotherapy/methods’.

Finally, according to statistical analysis, the MeSH term “depressive disorder, major/drug therapy” still had the greatest value of degree centrality during 2013–2018, similar to the two previous time-periods. In addition, six new major MeSH terms/MeSH subheadings were added to the nodes of the SNA during 2013–2018 (**Figure 7iii**, including “antidepressive agents/pharmacology”, “depressive disorder, treatment-resistant/drug therapy”, “depressive disorder, major/diagnosis”, “depression/drug therapy”, “depressive disorder, major/physiopathology”, and “antidepressive agents/adverse effects”, which were characterized by higher value of betweenness centrality (greater than the mean value of 5.917, **Table 5**). Furthermore, compared with the first and second time-periods of the SNA (**Figure 7**), a total of eight new emerging nodes (aqua square nodes in **Figure 7iii**), including “sulfides/therapeutic use”, “brain/drug effects”, “ketamine/therapeutic use”, “ketamine/pharmacology”, “depressive disorder, treatment-resistant/therapy”, “depression therapy”, “depressive disorder, major/epidemiology”, and “fluoxetine/therapeutic use”, were considered as emerging hotspots of drug therapy studies on MDD during 2013–2018.

## Discussion

Antidepressants are medications that can help relieve symptoms of depression, seasonal affective disorder, dysthymia, and mild chronic depression, as well as other conditions. These medications were first developed in 1950 and their use has become progressively more common (11), with approximately 70% of patients responding positively to at least one antidepressant (50). This study evaluated drug therapy studies on MDD in recent decades by exploring bibliometrics to reach the conclusion that publication related to the topic increased, with varying degrees of fluctuation, in the last 18 years since the beginning of the 21^st^ century. Moreover, after in-depth analysis, we observed that growth of publications during the third period of 2013-2018 slowed in comparison with the second period, and there was a slight decline in 2005, 2011, and 2016 (one in every period studied). In addition, consistent with the results of bibliometric analysis in other research fields, we found that the U.S. and England were the top two countries contributing the most to drug therapy studies on MDD (51, 52). This result is likely related to the fact that PubMed mainly includes articles written in English; that the first official language in these two countries is English; and that government support for scientific research funding and of influential scientists, among other factors, is prominent in both countries (52). However, based on the results of the strategic diagram, we observed that the contents of the theme clusters in each quadrant changed, to some extent, so that we have a preliminary understanding of theme trends from 2001 to 2018.

In the first period of 2001–2006, cluster 2 situated in quadrant III indicated that research on the functional mechanism of antidepressants in pathophysiology, neuroendocrinology, and neuro-biochemistry was immature and needed further study. We found that previous research qualitatively analyzed the degree of striatal dopamine and serotonin receptors/transporter occupancy by using technologies, including single photon emission computerized tomography and positron emission tomography to study the efficacy and side effect profile of antipsychotics, which provided evidence to uncover the pathophysiology mechanism of depressive disorder and other neuropsychiatric disorders (53). In addition, scholars propose that depressed patients suffer from a reduced number and/or function of glucocorticoid receptors (GR), and that antidepressants can exert therapeutic action by enhancing GR function expression (54). These findings further elucidate the biochemical and molecular mechanisms of MDD and lead to a new insight into the pathophysiology and treatment of the disease (54). However, due to the complex, and as of yet controversial, pathogenesis of MDD, mechanisms involved in its progression are only beginning to be elucidated. At the same time, studies on the development, mechanism of use, clinical application, and evaluation of antidepressant drugs against MDD are also increasing. Moreover, as antidepressant treatment based on the traditional monoamine theory results in delayed and high treatment inefficiency (55), scholars have been committed to the improvement of traditional antidepressants and to the discovery of new therapeutics that target the functional mechanisms of the disease.

Additionally, cluster 1 located in quadrant IV indicated that the influencing factors of therapeutic effect and clinical outcome of antidepressant agents were in immature stages of research development and needed further study. Previous reports had suggested that the knowledge and attitude of doctors toward the diagnostic criteria for depressive disorders (56), and the age, genotype, medical burden, physical and psychiatric symptoms, and alcohol consumption of the patients with MDD before treatment (57–59), could have a significant impact on the course and outcome of antidepressant medication. However, the genome, gender, medication compliance, and suicide ideation degree of the depressed patients also need to be taken into account when prescribing the different types and dosages of antidepressants (60–62). Subsequent research needs to integrate the above factors and to carry out in-depth discussion regarding the results.

During 2007–2012, cluster 2 located in quadrant III indicated that researchers focused on the topic of therapeutic effect and influencing factors of depressed patients after receiving psychotherapy alone, or combined with drug therapy. Psychological treatment (e.g., cognitive therapy and interpersonal psychotherapy), as the most common nonpharmacological intervention combined with pharmacotherapy, aims to enhance efficacy, to improve patients’ social functioning and QOL, and to prevent or delay recurrence. Previous studies have shown that the combination of psychological and pharmacological treatment is somewhat more effective than treatment with pharmacotherapy alone (63). Meanwhile, the results of a meta-analysis based on 18 studies and 1,838 subjects showed that a combined treatment was more effective than psychological treatment alone, especially for specific populations of older adults with chronic depression (64). However, these results need further verification by follow-up investigation in clinic. In addition, except for patient’s preference and compliance for treatment methods, the doctor-patient relationship, patients’ lifestyle, and other influencing factors need to be further explored (65). Clusters 2 and 3 were both the in quadrant III and had the same theme content; predictive studies on the effectiveness of antidepressant treatment based on brain imaging. Functional connectivity between the pregenual anterior cingulate cortex and the left amygdala is known to be negatively correlated with changes in antidepressant symptoms among patients with MDD (66). Scholars need to overcome current limitations where pre-treatment brain imaging can only explain what features are more likely to improve early symptoms, but not specific early and progressive changes in brain imaging of patients after drug therapy (67).

Anxiety is responsible for a reduction in the efficacy of antidepressant treatment. However, in contrast to the results of cluster 0 during 2001–2006 (68), the results of cluster 1 in the second phase of 2007-2012 demonstrated that escitalopram was better than paroxetine for highly depressed patients with comorbid anxiety symptoms (69). Another difference in the results of cluster 0 was that researchers not only focused on antidepressant only use (70), but were now more likely to evaluate and to prescribe antidepressants combined with antipsychotics (71). However, the evaluation of the specific treatment model, safety, tolerability and other factors of multi-drug combinations still need to be further improved.

During 2013–2018, cluster 1, with theme contents involved in the evaluation of the mechanism of action and therapeutic effect of new antidepressants, was located in quadrant I. Pearce et al. analyzed the mechanism of action of the new antidepressant agent vortioxetine and proposed it was an effective agent for the treatment of MDD (72). Afterwards, vortioxetine and agomelatine, as new antidepressant drugs, were respectively used for MDD patients with inadequate responses to SSRI/SNRI monotherapy. The authors were able to show that vortioxetine was safer and better tolerated by employing the clinical outcome indicators of changes in depression and anxiety symptoms, response and remission rates, QOL, productivity, and family functioning (73). In addition, for patients with MDD who are non-responsive or partially responsive to one or more antidepressants, researchers proposed that adjunctive therapy with atypical antipsychotics (e.g., aripiprazole) to be efficacious and well tolerated (74). The data indicate that, based on clinical empirical research, MDD patients who are difficult to treat have received extensive attention from researchers, which has resulted in better treatment options.

Furthermore, the theme contents of clusters 2–4, situated in quadrant III, were peripheral and undeveloped in the research field of drug therapy studies on MDD during the past 6 years of 2013–2018. Due to the demographic factors of patients, there may be differences in the effectiveness of drug treatments and onset of adverse reactions, leading to the progression or even deterioration of the disease (75). Therefore, researchers need to develop more antidepressants with rapid-onset that are safe and have fewer side effects. At present, ketamine, an antagonist of the N-methyl-D-aspartate family of glutamate receptors, has shown rapid antidepressant effectiveness in patients with MDD in clinic (76). However, given that ketamine’s specific mechanism of action is still in the theoretical hypothesis stage and that there are safety issues surrounding its use, the drug has not been used extensively as an antidepressant (77). Meanwhile, due to the existence of various complex pathways in the nervous system and negative feedback regulation mechanisms, research and development of single target therapy and mono-medication treatments have encountered a bottleneck. Thus, multi-target synergistic and combinatory drugs will become the main-line treatment in the future. In addition, genome-wide genotyping technology has made some progress in the study of genetic/biomarkers influencing the response and efficacy of antidepressant treatment, but due to the complexity of the pathogenesis of MDD, research still needs to be further improved (78). Moreover, scholars analyzed the mechanism of action and clinical therapeutic effect of antidepressant agents from two perspectives: the interaction between synaptic generation and neurogenesis (79), and the changes in the functional connections of multiple brain regions in patients with depressive disorder (80). However, relevant research conclusions have been based on the combination of multiple studies, and future research should generate a common and standardized strategy for data acquisition and statistical analysis to establish a scientifically and clinically useful and unified knowledge network by eliminating method bias as much as possible.

Finally, we can also conclude that the MeSH terms depressive disorder, major/drug therapy, and antidepressive agents/therapeutic use have the highest values of betweenness centrality, implying that they have the largest number of direct connections with other nodes, and that they are located at the core position within the SNAs. In other words, research on the functional mechanism of antidepressants; therapeutic effect and influencing factors of depressed patients after receiving single psychotherapy or combined with drug therapy; predictive studies on effectiveness of antidepressant treatment based on brain imaging; genetic/biomarkers that influence the response and effect of antidepressant treatment; antidepressant treatment on residual symptoms of depressed patients and improvement methods; and mechanism of action and clinical therapeutic effect of antidepressant agents are significant and potentially important academic issues in the field of MDD research. In addition, the new emerging hotspots during the three periods of 2001–2006, 2007–2012, and 2013–2018 studied should be viewed as a guide to finding new directions for research. Another emerging theme content was in regards to identification and medical intervention of MDD patients with suicide attempts, focusing on the analysis and extraction of the variables relevant to suicide attempts, and the impact of basic suicide tendency on the effect of antidepressants (8, 81). Specific medication intervention measures need to be further studied.

## Conclusions

According to analyze the publications in each period from 2001 to 2008, we can conclude that drug therapy studies on MDD have been a significant issue of general concern, but that progress has relatively slowed in recent six years. By integrating methods of bi-clustering, strategic diagram and SNAs analysis, the more significant findings of this study revealed the undeveloped theme clusters in recent decades, such as therapeutic effects of drug therapy in patients with MDD based on brain imaging, genetic/biomarkers affecting the response and efficacy of antidepressants, as well as mechanisms of adverse reaction of antidepressants. Furthermore, emerging MeSH terms/hotspots, such as antidepressive agents/administration & dosage, antidepressive agents/adverse effects, brain/drug effects also could provide medical staff, scientific researchers, and frontline educators with new research topics in the field of drug therapy studies on MDD.

### Limitation

There are several limitations in the current study: First, the data collection was only limited to papers that were published in PubMed which have led to publication bias to some extent. Second, considering that high-impact journals also have a high standard for the articles they include, the number of papers on the same research topic published in high-impact journals is limited, and many more may have been published in other relatively low-impact journals. Therefore, the extracted literature from different types of journals may contribute different weights to the formation of knowledge structure and the prediction of theme trends. Third, we carried out co-word analysis on the basis of extracting high-frequency MeSH terms/MeSH subheading by BICOMBS, which may have led to several new emerging subjects with low attention being missed due to a low word frequency that was below the threshold value of high-/low-frequency major MeSH terms/MeSH subheadings. Therefore, bibliometric analysis should be a dynamic process, and relevant results and conclusions should be further updated and improved through an increase in database sources and the extension of research time.

## Data Availability Statement

The raw data supporting the conclusions of this article will be made available by the authors, without undue reservation, to any qualified researcher.

## Author Contributions

GZ designed and corrected this paper. LD wrote this paper. YG collected and analyzed data. XS, CT, and CF collected data.

## Funding 

This project was supported by a grant from the Major Project of the Department of Science & Technology of Liaoning Province (2019JH8/10300019) and a grant from the Major Project of the Science and Technology Ministry in China (2017YFC0820200).

## Conflict of Interest

The authors declare that the research was conducted in the absence of any commercial or financial relationships that could be construed as a potential conflict of interest.

## References

[1] WHO Depression data fact sheet. Geneva: World Health Organization (2020). Available from: http://www.who.int/mediacentre/factsheets/fs369/en/ (Accessed July 08, 2020)

[2] Kessler RC Berglund P Demler O Jin R Koretz D Merikangas KR The epidemiology of major depressive disorder: results from the National Comorbidity Survey Replication (NCS-R). JAMA (2003) 289(23):3095–105. 10.1001/jama.289.23.3095 12813115

[3] Lam RW McIntosh D Wang J Enns MW Kolivakis T Michalak EE Canadian Network for Mood and Anxiety Treatments (CANMAT) 2016 Clinical Guidelines for the Management of Adults with Major Depressive Disorder: Section 1. Disease Burden and Principles of Care. Can J Psychiatry (2016) 61(9):510–23. 10.1177/0706743716659416 PMC4994789 27486151

[4] Huang Y Wang Y Wang H Liu Z Yu X Yan J Prevalence of mental disorders in China: a cross-sectional epidemiological study. Lancet Psychiatry (2019) 6(3):211–24. 10.1016/s2215-0366(18)30511-x 30792114

[5] Woo YS Rosenblat JD Kakar R Bahk W-M McIntyre RS Cognitive Deficits as a Mediator of Poor Occupational Function in Remitted Major Depressive Disorder Patients. Clin Psychopharmacol Neurosci (2016) 14(1):1–16. 10.9758/cpn.2016.14.1.1 26792035 PMC4730927

[6] Gonda X Fountoulakis KN Kaprinis G Rihmer Z Prediction and prevention of suicide in patients with unipolar depression and anxiety. Ann Gen Psychiatry (2007) 6:23. 10.1186/1744-859X-6-23 17803824 PMC2031887

[7] Chen H-M Hou S-Y Yeh Y-C Chang C-Y Yen J-Y Ko C-H Frontal function, disability and caregiver burden in elderly patients with major depressive disorder. Kaohsiung J Med Sci (2010) 26(10):548–54. 10.1016/S1607-551X(10)70084-X PMC11915952 20950780

[8] Bingham KS Rothschild AJ Mulsant BH Whyte EM Meyers BS Banerjee S The Association of Baseline Suicidality With Treatment Outcome in Psychotic Depression. J Clin Psychiatry (2016) 78(8):1149–54. 10.4088/JCP.16m10881 28445632

[9] Murray CJL Barber RM Foreman KJ Abbasoglu Ozgoren A Abd-Allah F Abera SF Global, regional, and national disability-adjusted life years (DALYs) for 306 diseases and injuries and healthy life expectancy (HALE) for 188 countries, 1990-2013: quantifying the epidemiological transition. Lancet (2015) 386(10009):2145–91. 10.1016/S0140-6736(15)61340-X PMC4673910 26321261

[10] Mathers CD Loncar D Projections of global mortality and burden of disease from 2002 to 2030. PloS Med (2006) 3(11):e442. 10.1371/journal.pmed.0030442 17132052 PMC1664601

[11] Lieberman J History of the use of antidepressants in primary care. J Clin Psychiatry (2003) 5(Suppl 7):6–10.

[12] Karp JF Skidmore E Lotz M Lenze E Dew MA Reynolds CF Use of the Late-Life Function and Disability Instrument to Assess Disability in Major Depression. J Am Geriatr Soc (2009) 57(9):1612–9. 10.1111/j.1532-5415.2009.02398.x PMC2854008 19682111

[13] Blier P The pharmacology of putative early-onset antidepressant strategies. Eur Neuropsychopharmacol (2003) 13(2):57–66. 10.1016/s0924-977x(02)00173-6 12650947

[14] Andrade C Relative Efficacy and Acceptability of Antidepressant Drugs in Adults With Major Depressive Disorder: Commentary on a Network Meta-Analysis. J Clin Psychiatry (2018) 79(2):18f12254. 10.4088/JCP.18f12254 29718600

[15] Bengalorkar G Bhuvana K Raju GN N S. 7. A novel atypical antidepressant drug: Agomelatine - A review. Int J Pharmaceut Biomed Res (IJPBR) (2010) 1(3):113–16.

[16] Lee G Bae H Therapeutic effects of phytochemicals and medicinal herbs on depression. BioMed Res Int (2017) 2017:6596241. 10.1155/2017/6596241 28503571 PMC5414506

[17] Xu Y Wang C J Klabnik J O’Donnell J M Novel therapeutic targets in depression and anxiety: antioxidants as a candidate treatment. Curr Neuropharmacol (2014) 12(2):108–19. 10.2174/1570159X11666131120231448 PMC3964743 24669206

[18] Wiles NJ Thomas L Turner N Garfield K Kounali D Campbell J Long-term effectiveness and cost-effectiveness of cognitive behavioural therapy as an adjunct to pharmacotherapy for treatment-resistant depression in primary care: follow-up of the CoBalT randomised controlled trial. Lancet Psychiatry (2016) 3(2):137–44. 10.1016/S2215-0366(15)00495-2 26777773

[19] Goldstein-Piekarski AN Staveland BR Ball TM Yesavage J Korgaonkar MS Williams LM Intrinsic functional connectivity predicts remission on antidepressants: a randomized controlled trial to identify clinically applicable imaging biomarkers. Transl Psychiatry (2018) 8(1):57. 10.1038/s41398-018-0100-3 29507282 PMC5838245

[20] Ma D Zhang Z Zhang X Li L Comparative efficacy, acceptability, and safety of medicinal, cognitive-behavioral therapy, and placebo treatments for acute major depressive disorder in children and adolescents: a multiple-treatments meta-analysis. Curr Med Res Opin (2014) 30(6):971–95. 10.1185/03007995.2013.860020 24188102

[21] Nishimura A Aritomi Y Sasai K Kitagawa T Mahableshwarkar AR Randomized, double-blind, placebo-controlled 8-week trial of the efficacy, safety, and tolerability of 5, 10, and 20 mg/day vortioxetine in adults with major depressive disorder. Psychiatry Clin Neurosci (2018) 72(2):64–72. 10.1111/pcn.12565 28858412

[22] Pritchard A Statistical Bibliography or Bibliometrics? J Doc (1969) 25(4):348–9.

[23] Guler AT Waaijer CJF Palmblad M Scientific workflows for bibliometrics. Scientometrics (2016) 107(2):385–98. 10.1007/s11192-016-1885-6 PMC4833826 27122644

[24] Dalpé R Bibliometric analysis of biotechnology. Scientometrics (2002) 55(2):189–213. 10.1023/a:1019663607103

[25] Nederhof AJ Bibliometric monitoring of research performance in the social sciences and the humanities: A review. Scientometrics (2006) 66(1):81–100. 10.1371/journal.pmed.0040040

[26] Thompson DF Walker CK descriptive A and historical review of bibliometrics with applications to medical sciences. Pharmacotherapy (2015) 35(6):551–9. 10.1002/phar.1586 25940769

[27] Ronda-Pupo GA Guerras-Martin LÁ Dynamics of the evolution of the strategy concept 1962-2008: a co-word analysis. Strat Manage J (2012) 33(2):162–88. 10.1002/smj.948

[28] Landis JR Koch GG The measurement of observer agreement for categorical data. Biometrics (1977) 33(1):159–74. 10.2307/2529310 843571

[29] Hirsch JE An index to quantify an individual’s scientific research output. Proc Natl Acad Sci USA (2005) 102(46):16569–72. 10.1073/pnas.0507655102 PMC1283832 16275915

[30] Guan J Gao X Exploring the h-Index at Patent Level. J Assoc Inf Sci Technol (2009) 60(1):35–40. 10.1002/asi.20954

[31] Gray R HE Thompson DR Journal editors and their h-index. J Adv Nurs (2016) 73(9):2031–4. 10.1111/jan.13070 27400154

[32] Raan AFJV Comparison of the Hirsch-index with standard bibliometric indicators and with peer judgment for 147 chemistry research groups. Scientometrics (2006) 67(3):491–502. 10.1556/Scient.67.2006.3.10

[33] La Torre G Sciarra I Chiappetta M Monteduro A New bibliometric indicators for the scientific literature: an evolving panorama. Clin Ter (2017) 168(2):e65–71. 10.7417/CT.2017.1985 28383616

[34] Li F Li M Guan P Ma S Cui L Mapping publication trends and identifying hot spots of research on Internet health information seeking behavior: a quantitative and co-word biclustering analysis. J Med Internet Res (2015) 17(3):e81. 10.2196/jmir.3326 25830358 PMC4390616

[35] Hartigan JA Direct Clustering of a Data Matrix. Publ Am Stat Assoc (1972) 67(337):7. 10.1080/01621459.1972.10481214

[36] Oghabian A Kilpinen S Hautaniemi S Czeizler E Biclustering methods: biological relevance and application in gene expression analysis. PloS One (2014) 9(3):e90801. 10.1371/journal.pone.0090801 24651574 PMC3961251

[37] Lu K Yu S Yu M Sun D Huang Z Xing H Bibliometric Analysis of Tumor Immunotherapy Studies. Med Sci Monit (2018) 24:3405–14. 10.12659/MSM.910724 PMC5994141 29790485

[38] Karypis Lab gCLUTO-Graphical Clustering Toolkit. Minneapolis: Karypis Lab. (2003). Available from: http://glaros.dtc.umn.edu/gkhome/cluto/gcluto/download. (Accessed November 02, 2019).

[39] Law J Bauin S Courtial J-P Whittaker J Policy and the mapping of scientific change A co-word analysis of research into environmental acidification. Scientometrics (1988) 14(3-4):251–64. 10.1007/bf02020078

[40] Callow M Courtial JP Turner WA From translations to problematic networks An introduction to co-word analysis. Soc Sci Inf (1983) 22(2):191–235. 10.1177/053901883022002003

[41] Dehdarirad T Villarroya A Barrios M Research trends in gender differences in higher education and science: a co-word analysis. Scientometrics (2014) 101(1):273–90. 10.1007/s11192-014-1327-2

[42] Gan J Cai Q Galer P Ma D Chen X Huang J Mapping the knowledge structure and trends of epilepsy genetics over the past decade: A co-word analysis based on medical subject headings terms. Med (Baltimore) (2019) 98(32):e16782. 10.1097/MD.0000000000016782 PMC6709143 31393404

[43] Callon M Courtial JP Laville F Co-word analysis as a tool for describing the network of interactions between basic and technological research The case of polymer chemsitry. Scientometrics (1991) 22(1):155–205. 10.1007/BF02019280

[44] Wasserman S Faust K Social network analysis: methods and applications. Cambridge: Cambridge University Press (1994).

[45] Bright C Conceptualizing Deviance: A Cross-Cultural Social Network Approach to Comparing Relational and Attribute Data. London: Rowman & Littlefield (2016).

[46] Valente TW Fujimoto K Palmer P Tanjasiri SP A network assessment of community-based participatory research: linking communities and universities to reduce cancer disparities. Am J Public Health (2010) 100(7):1319–25. 10.2105/AJPH.2009.171116 PMC2882399 20466964

[47] Sadria M Karimi S Layton AT Network centrality analysis of eye-gaze data in autism spectrum disorder. Comput Biol Med (2019) 111:103332. 10.1016/j.compbiomed.2019.103332 31276943

[48] Freeman LC Centrality in Social Networks’ Conceptual Clarification. Soc Networks (1979) 1(3):215–39. 10.1016/0378-8733(78)90021-7

[49] Günther R Georgi M Kärst T Automatic Knowledge Acquisition from MEDLINE. Methods Inf Med (1993) 32(2):120–30. 10.1055/s-0038-1634904 8321130

[50] Ananth J Choosing the right antidepressant. Psychiatr J Univ Ottawa: Rev Psychiatr L’Universite D’Ottawa (1983) 8(1):20. 6346364

[51] Brown T Gutman SA Ho Y-S Fong KNK A bibliometric analysis of occupational therapy publications. Scand J Occup Ther (2018) 25(1):1–14. 10.1080/11038128.2017.1329344 28508696

[52] Saunders TFC Rymer BC McNamara KJ A global bibliometric analysis of otolaryngology: Head and neck surgery literature. Clin Otolaryngol (2017) 42(6):1338–42. 10.1111/coa.12910 28561944

[53] Kasper S Tauscher J Willeit M Stamenkovic M Neumeister A Küfferle B Receptor and transporter imaging studies in schizophrenia, depression, bulimia and Tourette’s disorder–implications for psychopharmacology. World J Biol Psychiatry (2002) 3(3):133–46. 10.3109/15622970209150614 12478878

[54] Pariante CM Miller AH Glucocorticoid receptors in major depression: relevance to pathophysiology and treatment. Biol Psychiatry (2001) 49(5):391–404. 10.1016/s0006-3223(00)01088-x 11274650

[55] Tylee A Walters P Onset of action of antidepressants. BMJ (2007) 334(7600):911–2. 10.1136/bmj.39197.619190.80 PMC1865459 17478791

[56] Lublin HK Nielsen UJ Vittrup P Bach-Dal C Larsen JK Diagnosis and treatment of depression in general practice. A questionnaire study. Ugeskrift Laeger (2002) 164(26):3440–4. 12119755

[57] Goldstein BI Schaffer A Levitt A Zaretsky A Joffe RT Wesson V Depressive symptoms and alcohol consumption among nonalcoholic depression patients treated with desipramine. Can J Psychiatry Rev Can Psychiatr (2004) 49(12):859–62. 10.1177/070674370404901210 15679210

[58] Adeoye OM Ferrell RE Kirshner MA Mulsant BH Seligman K Begley AE alpha1-acid glycoprotein in late-life depression: relationship to medical burden and genetics. J Geriatric Psychiatry Neurol (2003) 16(4):235–9. 10.1177/0891988703258321 14653433

[59] Arias B Serretti A Lorenzi C Gastó C Catalán R Fañanás L Analysis of COMT gene (Val 158 Met polymorphism) in the clinical response to SSRIs in depressive patients of European origin. J Affect Disord (2006) 90(2-3):251–6. 10.1016/j.jad.2005.11.008 16356553

[60] Su Y-A Li J-T Dai W-J Liao X-M Dong L-C Lu T-L Genetic variation in the tryptophan hydroxylase 2 gene moderates depressive symptom trajectories and remission over 8 weeks of escitalopram treatment. Int Clin Psychopharmacol (2016) 31(3):127–33. 10.1097/YIC.0000000000000115 26745768

[61] Tokuoka H Nishihara M Fujikoshi S Yoshikawa A Kuga A Predicting treatment outcomes of major depressive disorder by early improvement in painful physical symptoms: A pooled analysis of double-blind, placebo-controlled trials of duloxetine. Neuropsychiatr Dis Treat (2017) 13:2457–67. 10.2147/NDT.S143093 PMC5626379 29026309

[62] Rihmer Z Gonda X Prevention of depression-related suicides in primary care. Psychiatr Hung (2012) 27(2):72–81. 22700618

[63] Pampallona S Bollini P Tibaldi G Kupelnick B Munizza C Combined pharmacotherapy and psychological treatment for depression: a systematic review. Arch Gen Psychiatry (2004) 61(7):714–9. 10.1001/archpsyc.61.7.714 15237083

[64] Cuijpers P van Straten A Warmerdam L Andersson G Psychotherapy versus the combination of psychotherapy and pharmacotherapy in the treatment of depression: a meta-analysis. Depression Anxiety (2009) 26(3):279–88. 10.1002/da.20519 19031487

[65] Peeters F Huibers M Roelofs J van Breukelen G Hollon SD Markowitz JC The clinical effectiveness of evidence-based interventions for depression: a pragmatic trial in routine practice. J Affect Disord (2013) 145(3):349–55. 10.1016/j.jad.2012.08.022 22985486

[66] Salvadore G Cornwell BR Sambataro F Latov D Colon-Rosario V Carver F Anterior cingulate desynchronization and functional connectivity with the amygdala during a working memory task predict rapid antidepressant response to ketamine. Neuropsychopharmacology (2010) 35(7):1415–22. 10.1038/npp.2010.24 PMC2869391 20393460

[67] Hou Z Kong Y He X Yin Y Zhang Y Yuan Y Increased temporal variability of striatum region facilitating the early antidepressant response in patients with major depressive disorder. Prog Neuropsychopharmacol Biol Psychiatry (2018) 85:39–45. 10.1016/j.pnpbp.2018.03.026 29608926

[68] Wagstaff AJ Cheer SM Matheson AJ Ormrod D Goa KL Spotlight on paroxetine in psychiatric disorders in adults. CNS Drugs (2002) 16(6):425–34. 10.2165/00023210-200216060-00006 12027788

[69] Chauvet-Gélinier JC Efficacy of escitalopram vs paroxetine on severe depression with associated anxiety: data from the “Boulenger” study. L’Encephale (2010) 36(5):425–32. 10.1016/j.encep.2010.08.001 21035633

[70] Dichter GS Tomarken AJ Freid CM Addington S Shelton RC Do venlafaxine XR and paroxetine equally influence negative and positive affect? J Affect Disord (2005) 85(3):333–9. 10.1016/j.jad.2004.10.007 15780704

[71] Blier P Gobbi G Turcotte JE de Montigny C Boucher N Hébert C Mirtazapine and paroxetine in major depression: a comparison of monotherapy versus their combination from treatment initiation. Eur Neuropsychopharmacol (2009) 19(7):457–65. 10.1016/j.euroneuro.2009.01.015 19345072

[72] Pearce EF Murphy JA Vortioxetine for the treatment of depression. Ann Pharmacother (2014) 48(6):758–65. 10.1177/1060028014528305 24676550

[73] Frampton JE Vortioxetine: A Review in Cognitive Dysfunction in Depression. Drugs (2016) 76(17):1675–82. 10.1007/s40265-016-0655-3 27807822

[74] Jon DI Kim DH Seo HJ Kwon YJ Kim MD Yang JC Augmentation of aripiprazole for depressed patients with an inadequate response to antidepressant treatment: a 6-week prospective, open-label, multicenter study. Clin Neuropharmacol (2014) 36(5):157–61. 10.1097/WNF.0b013e3182a31f3d 24045606

[75] Germain A Kupfer DJ Circadian rhythm disturbances in depression&nbsp. Hum Psychopharmacol (2008) 23(7):571–85. 10.1002/hup.964 PMC2612129 18680211

[76] Singh JB Fedgchin M Daly E Xi L Melman C De Bruecker G Intravenous Esketamine in Adult Treatment-Resistant Depression: A Double-Blind, Double-Randomization, Placebo-Controlled Study. Biol Psychiatry (2016) 80(6):424–31. 10.1016/j.biopsych.2015.10.018 26707087

[77] Miller OH Moran JT Hall BJ Two cellular hypotheses explaining the initiation of ketamine’s antidepressant actions: Direct inhibition and disinhibition. Neuropharmacology (2016) 100:17–26. 10.1016/j.neuropharm.2015.07.028 26211972

[78] McMahon FJ Clinically Useful Genetic Markers of Antidepressant Response: How Do We Get There From Here? Am J Psychiatry (2015) 172(8):697–9. 10.1176/appi.ajp.2015.15050644 26234589

[79] Bambico FR Belzung C Novel insights into depression and antidepressants: a synergy between synaptogenesis and neurogenesis? Curr Topics Behav Neurosci (2013) 15:243–91. 10.1007/7854_2012_234 23271325

[80] Brakowski J Spinelli S Dörig N Bosch OG Manoliu A Holtforth MG Resting state brain network function in major depression - Depression symptomatology, antidepressant treatment effects, future research. J Psychiatr Res (2017) 92:147–59. 10.1016/j.jpsychires.2017.04.007 28458140

[81] Popovic D Vieta E Azorin JM Angst J Bowden CL Mosolov S Suicide attempts in major depressive episode: evidence from the BRIDGE-II-Mix study. Bipolar Disord (2015) 17(7):795–803. 10.1111/bdi.12338 26415692

